# Novel Sex-Specific Genes and Diverse Interspecific Expression in the Antennal Transcriptomes of Ithomiine Butterflies

**DOI:** 10.1093/gbe/evae218

**Published:** 2024-10-07

**Authors:** Francesco Cicconardi, Billy J Morris, Jacopo Martelossi, David A Ray, Stephen H Montgomery

**Affiliations:** School of Biological Sciences, Bristol University, 24 Tyndall Ave, Bristol BS8 1TQ, UK; Department of Zoology, University of Cambridge, Downing Street, Cambridge CB2 3EJ, UK; Department of Biological Geological and Environmental Science, University of Bologna, Via Selmi 3, 40126 Bologna, Italy; Department of Biological Sciences, Texas Tech University, Lubbock, TX 79409, USA; School of Biological Sciences, Bristol University, 24 Tyndall Ave, Bristol BS8 1TQ, UK

**Keywords:** antennal transcriptomics, chemosensory genes, comparative genomics, sensory adaptation, sexual dimorphism

## Abstract

The olfactory sense is crucial for organisms, facilitating environmental recognition and interindividual communication. Ithomiini butterflies exemplify this importance not only because they rely strongly on olfactory cues for both inter- and intra-sexual behaviors, but also because they show convergent evolution of specialized structures within the antennal lobe, called macroglomerular complexes (MGCs). These structures, widely absent in butterflies, are present in moths where they enable heightened sensitivity to, and integration of, information from various types of pheromones. In this study, we investigate chemosensory evolution across six Ithomiini species and identify possible links between expression profiles and neuroanatomical. To enable this, we sequenced four new high-quality genome assemblies and six sex-specific antennal transcriptomes for three of these species with different MGC morphologies. With extensive genomic analyses, we found that the expression of antennal transcriptomes across species exhibit profound divergence, and identified highly expressed ORs, which we hypothesize may be associated to MGCs, as highly expressed ORs are absent in *Methona*, an Ithomiini lineage which also lacks MGCs. More broadly, we show how antennal sexual dimorphism is prevalent in both chemosensory genes and non-chemosensory genes, with possible relevance for behavior. As an example, we show how lipid-related genes exhibit consistent sexual dimorphism, potentially linked to lipid transport or host selection. In this study, we investigate the antennal chemosensory adaptations, suggesting a link between genetic diversity, ecological specialization, and sensory perception with the convergent evolution of MCGs. Insights into chemosensory gene evolution, expression patterns, and potential functional implications enhance our knowledge of sensory adaptations and sexual dimorphisms in butterflies, laying the foundation for future investigations into the genetic drivers of insect behavior, adaptation, and speciation.

**Significance**

Understanding how organisms detect odors is crucial as it influences environmental interactions and communication. In this study, we assemble four new genomes of the hyper-diverse tribe of Ithomiini and explore sex and species differences in antennal gene expression. This revealed significant interspecies variation and olfactory receptors which may be associated with the convergent evolution of macroglomeruli in this tribe, providing candidate new pheromone receptors. These findings shed light on the genetic mechanisms behind olfactory adaptation and sensory evolution, enhancing our knowledge of how genetic diversity influences behavior and ecological specialization in butterflies.

## Introduction

Insects constitute one of the planet's most successful and diverse eukaryotic classes, accounting for roughly 50% of all land-dwelling species (Mora et al. 2011), across an astonishingly broad spectrum of environments. Research into the anatomical, physiological, and behavioral facets of this diversity has demonstrated the evolutionary malleability and importance of insect sensory systems, including chemoreception (Missbach et al. 2014). A significant emphasis has been placed on the identification and functional characterization of olfactory receptors (Yan et al. 2020), as well as the neural circuits in which they are expressed, and the odor-driven behaviors that they govern (Amin and Lin 2019). While olfactory reception and circuit evolution has been linked to some cases of ecological speciation (Olsson et al. 2006; Prieto-Godino et al. 2017; Auer et al. 2020, 2022), the ecological selection pressures shaping olfactory evolution often remains challenging to pinpoint, particularly given the diversity of receptor types and associated gene families.

There are two primary categories of insect olfactory receptors: odorant receptors (ORs) (Vosshall et al. 1999) and ionotropic receptors (IRs) (Benton et al. 2009). OR genes encode for 7-transmembrane (7-TM) proteins, which create a homodimer of a heterodimer odor-gated ion channel through a combination of ligand-specific (“tuning”) receptor subunits (ORx) and a co-receptor, Orco (Mika and Benton 2021). OR proteins can identify odorants in lymph fluid, transforming chemical cues into neuroelectric signals and transmitting them to the central nervous system, thereby influencing insect behavior (Fleischer et al. 2018). IRs represent a markedly diverse subset of ionotropic glutamate receptors (iGluRs) (Benton et al. 2009). Predominantly, iGluRs bind the excitatory neurotransmitter glutamate and are instrumental in synaptic communication within the brain (Yelshanskaya et al. 2014). In contrast, IRs had been hypothesized to be the most ancient arthropod chemoreceptors, dating back to the Protostomia ancestor (Eyun et al. 2017). They have a primary and extensive presence in peripheral sensory systems, serving various functions including chemosensation, thermosensation, hygrosensation (Van Giesen and Garrity 2022), and potentially nonolfactory functions, such as mechanosensation (Senthilan et al. 2012). Within IRs, “antennal” IRs are conserved throughout insects and function in olfaction, thermosensation, and hygrosensation, while “divergent” IRs are expressed in peripheral and internal gustatory neurons and contribute to taste and food assessment (Croset et al. 2010). Structurally, they are similar to iGluRs, and a functional ion channel is formed by three-pass transmembrane of a homodimer of a heterodimer of IR subunits. In most cases, IRs consist of specific tuning receptors for different stimuli, alongside one or two broadly expressed co-receptors (Abuin et al. 2011).

Odorant sensory neurons (OSNs), located in the antennae, directly detect odor molecules in the environment before sending signals to the antennal lobe, the primary olfactory processing center in the insect brain. Studying sensory neurons allows us to analyze the initial response to different odor stimuli without interference from higher-order processing. OSNs are also often highly specific to particular compounds, which can facilitate more precise studies of differences in perception in a more granular level. In that context, comparative studies on the gene expression of OSNs across different species can provide valuable insights into the evolution and diversity of olfactory systems and perception, revealing common principles as well as species-specific adaptations. The canonical view is that the majority of olfactory sensory neurons (OSNs) generally express a pair of distinct ligand-selective ORs or IRs, in a one-receptor-to-one-neuron organization: a distinctive “tuning” receptor designed to detect specific ligands or odorants. This has recently been challenged by data showing that some neurons co-express multiple chemosensory receptors in *Aedes aegypti* (Herre et al. 2022; Task et al. 2022), however, how generally this occurs is yet to be investigated. Ligand-selective receptors are always associated with specific co-receptors (Orco for ORs, and either IR8a or IR25a for IRs), which do not recognize compounds but instead are needed to form heteromeric complexes with tuning ligand binding receptors (Schmidt and Benton 2020). Antennal lymph fluid also contains abundant secreted proteins and proteoglycans (Schmidt and Benton 2020), which influence the intrinsic physicochemical traits of the odors. Among these lymphatic proteins are the odorant-binding proteins (OBPs), which encode for small globular and soluble proteins. OBPs allow hydrophobic airborne odorants to dissolve into the lymph fluid and bind odorant compounds with different degrees of affinity and specificity, shuttling them to the underlying receptors in the form of monomers and/or homodimers (Leal 2013; Larter et al. 2016).

The diversity and variable evolutionary rates of olfactory receptors suggest an intimate link to ecological variation and species selection regimes. Lepidopterans have frequently been utilized as models among various insect species to explore the influence of ecological variability on the evolution of olfactory systems. Within them, Ithomiini butterflies offer an interesting system due to their chemical defenses, reliance on chemical communication, and interspecific interactions. They are one of the most specious tribes of Neotropical butterflies with 393 species and dominate butterfly communities in Neotropical forests (Beccaloni 1997b, 1997a). Their chemical defenses are primarily derived from pyrrolizidine alkaloids (PAs) from specific host plants, either obtained as larvae or through adult foraging and male-to-female provision through the spermatophore (Brown Jr 1985; Masters 1992; Massuda and Trigo 2009). Males generally have a stronger attraction toward these plants compared to females, but in some species, they also function as female attractants at short ranges and male repellents at long range, especially in species where males establish territorial dominance defending resource patches (Thomas E Pliske 1975b, 1975a). The complexity of these chemically driven behaviors is an indicator of a strong sexual dimorphism within adults in relation to PA sources, both in chemical defenses and pheromones, suggesting that this ecological context might have led to specific olfactory adaptations.

**Fig. 1.**
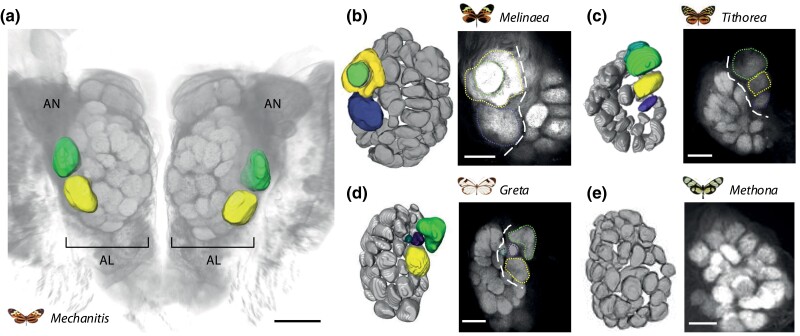
Antennal lobe morphology in Ithomiini. a) Volume rendering of a *M. polymnia* central brain, showing the position of the antennal lobes (ALs) and antennal nerves (ANs). The two enlarged glomeruli that form the MGC in this species are segmented and colored. Other glomeruli can be seen as smaller spherical shapes within the AL. MGC glomeruli are typically enlarged, but may occur together with associated, smaller “accessory” glomeruli, as part of a morphologically distinct cluster at the base of the AN (see also Montgomery and Ott 2015; Morris et al. 2021). *Mechanitis* belongs to the subtribe Mechanitina which generally show an MGC of two large glomeruli and sexual dimorphism. b to e) 3D segmentations (left) and example *x–y* confocal images of antennal lobe morphology for b) *Melinaea*, which belongs to the Melinaeina which show a MGC of three glomeruli, two of which are particularly large, and are sexually dimorphic; c) *Tithorea*, which belongs to the Tithoreina which appear to show a MGC of three or four glomeruli; d) *Greta*, which belongs to the Godryidina which typically show a MGC of up to four glomeruli, of variable sizes and variable levels of sexual dimorphism; and e) *Methona*, which belongs to the Methonina which show an absence of a MGC, no enlarged glomeruli or sexual dimorphism. All images were produced using immunohistochemical stains against an anti-SYNORF1 antibody, with confocal imaging, and adapted or reproduced following Morris et al. (2021), which performed an in-depth comparative study of antennal lobe morphologies across Ithomiini, including quantitative analysis of MGC glomerulus sizes. Glomeruli are color-coded from the base of the AN, including smaller “accessory” glomeruli associated with the MGC. Color-coding does not imply homology. The dashed lines within the photos demarcates the MGC. Scale bar in a) is 100 μm, b to e) its 50μm.

These adaptations likely lie in the antennae, antennal receptor cells, and downstream olfactory processing areas. In general, however, diurnal butterflies lack the striking specializations observed in moths. The antennal lobe of all lepidoptera is generally formed by ∼60 to 70 morphological units, called glomeruli, which are each composed of axon terminals from antennal sensory neurons expressing the same olfactory receptor (Rospars 1983; Hansson and Stensmyr 2011; Carlsson et al. 2013). In many moths, the antennal lobe of males is characterized by macroglomerular complexes (MGCs), specialized structures within the antennal lobe composed of interconnected and enlarged glomeruli that often respond to pheromones (Koontz and Schneider 1987; Sung et al. 2017; Williams et al. 2022). MGCs enable the integration of information from various types of pheromones and their associated odorants, facilitating precise and refined responses to specific cues. In butterflies, likely due to their increased reliance on visual cues, these structures are widely absent and were likely lost at the origin of the superfamily (Morris et al 2021). However, within the Ithomiini group, analogous structures to MGCs have reemerged through convergent evolution (Montgomery and Ott 2015; Morris et al. 2021). In ithomiines, the composition and size of these structures are highly variable across species and in many cases exhibit a degree of sexual dimorphism, with certain MGC being larger in males than in females, although in some species the enlarged glomeruli are shared between sexes (Morris et al. 2021) (Fig. 1). In contrast, in one genus, *Methona*, antennal glomeruli are of uniform size and lack enlarged or dimorphic glomeruli, suggesting a secondary loss of MGCs in this lineage (Morris et al. 2021) (Fig. 1e). *Methona* has a distinct mating strategy, with males engaging in aggressive aerial “take downs” of females (Pliske 1975a), a strategy which relies less on olfactory signaling to females than other Ithomiini genera (Brown Jr 1985; Brown 1987; McClure et al. 2019), which potentially explains their lack of a MGC. These patterns of MGC variation strongly suggests a unique, derived, and heightened reliance on olfactory reception in this tribe of butterflies, but with species-specific divergences in the presence/absence of traits, offering a case study to understand the evolution of new neurosensory traits at a molecular and ecological level. Because each antennal lobe glomerulus is innervated by OSNs expressing the same OR, the reemergence and diversity of an MGC in ithomiines imply that these anatomical traits should be mirrored by patterns of derived OR expression in the antennal OSNs. Specifically, we hypothesize that enlarged glomeruli should be associated with highly expressed ORs, while species lacking MGCs should show an absence of such outliers.

Our aim here is to test these predictions and to explore the genetic basis of these sexual dimorphism/adaptations, by sequencing the genomes of four Ithomiini species—*Mechanitis polymnia, Tithorea harmonia, Methona confusa*, and *Greta morgane—*which, together with published data for a fifth ithomiine genus, *Melinaea (Gauthier*et al*. 2023)*, represent deep divisions within the ithomiine phylogeny and variable ecologies and neuromorphologies. Beyond these specific aims, these genomic resources will be broadly useful for assessing ithomiine phylogenetics and patterns of molecular evolution. Here, we focus on using these resources to characterize and manually curate the antennal chemosensory receptors and OBPs, alongside data from 11 other species from closely and more distantly related nymphalid butterflies. We use this data to test for ithomiine-specific signatures of gene family evolution and selection and divergent patterns of receptor expression using antennal transcriptomes between sexes and species in three Ithomiini butterflies. We find that in Ithomiini butterflies, (i) olfactory innovations did not involve particularly major antennal chemosensory gene (ACG) expansion events; (ii) *neuro-dimorphic species* have over- and sex-biased ACG expression, mirroring anatomical differences among sex and species, with the number of OR expression outliers directly reflecting the number of MGC glomeruli in each species; and (iii) there is a strong transcriptomic diversity among species, possibly reflecting the different ecological niches in which these species are adapted.

## Results

### New Genomic Resources for Ithomiini Butterflies

We assembled new genomic resources for four ithomiine species: *M. polymnia, T. harmonia, M. confusa*, and *G. morgane.* A total of 180 Gb of linked-reads on average per sample were generated, which resulted in high coverage per sample, which was strategically downsampled to optimize contiguity and completeness (supplementary table S1, Supplementary Material online). The resulting assembly size (supplementary table S1, Supplementary Material online) is close to the estimated genome size for each species (500 Mb on average), with high contiguity per assembly (average N50 ∼ 4.6 Mb per sample; supplementary fig. S1 and table S1, Supplementary Material online). Completeness is very high in all four species (∼97% of single-copy BUSCO genes, 1% missing, with 1% of duplicated genes; supplementary table S1, Supplementary Material online), with a gene content of ∼20k on average (for more details, please see supplementary table S1 and figs. S2 to S4, Supplementary Material online).

To facilitate broader evolutionary analyses, we combined our Ithomiini data with published genomic data for species representing outgroup lineages. As anticipated, genome size and contiguity were influenced by the content of repeats (supplementary fig. S3, Supplementary Material online), with retroelements (SINE + LTR + LINE) and DNA transposons particularly affecting genome size (supplementary fig. S4, Supplementary Material online). Danainae are generally characterized by a lower proportion of repeats (50 Mb; 16% on average) compared with the other species in which repeat proportions always greater than 21% were found. On average, compared with Heliconiini, a well-studied tribe of nymphalid butterflies, Ithomiini have slightly lower repeat content, 26% (105 Mb) versus 34% (132 Mb), although this correlates with the larger genome size of Ithomiini (∼500 Mb). Ithomiini have ∼8 Mb (∼9% of the total TE content) of rolling circles (*Helitrons*), similar to other butterflies, but ∼8 times less compared with Heliconiini (on average ∼62 Mb; ∼48% of the total TE content), which seem to be particularly expanded in the latter. Within Ithomiini, *Melinaea* spp. show a particularly high number of rolling circles, with ∼15 Mb, six times more compared with other species. This enrichment seems to be expanded recently, as shown by the kimura 2-parameter (K2P) distances, calculated taking into account both transitions and transversions, assuming transitions occur more frequently than transversions (supplementary table S1 and fig. S5, Supplementary Material online).

**Fig. 2.**
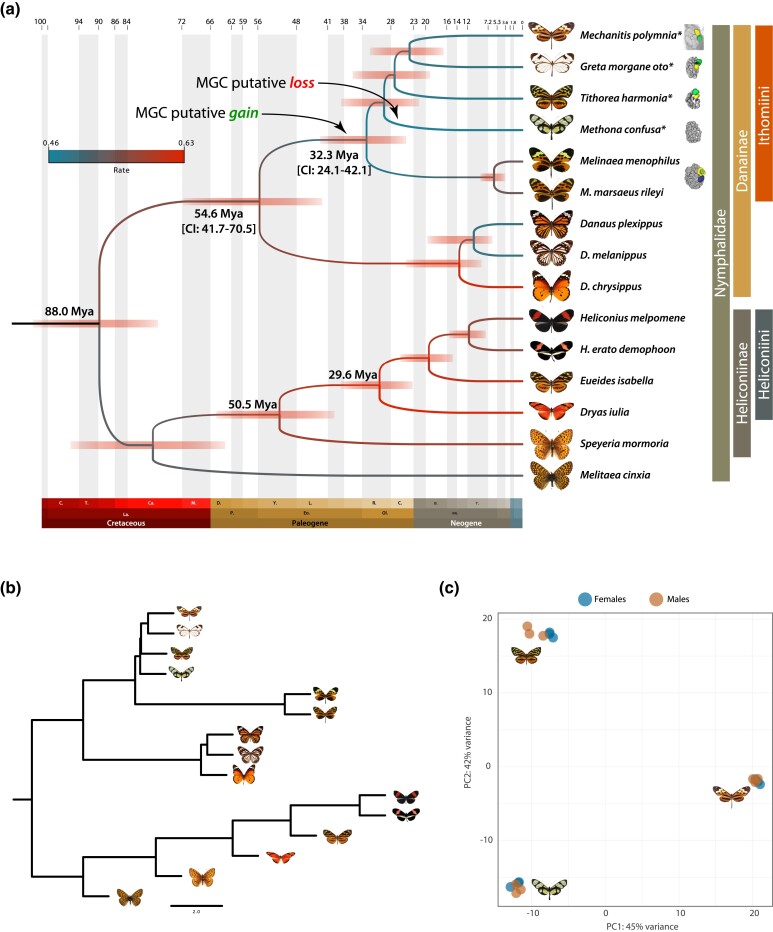
Phylogeny of Ithomiini butterflies and antennal transcriptomic diversity. a) Dated phylogeny of Ithomiini butterflies in the context of other nymphalids. The Ithomiini stem is dated between 24 and 42 Mya, overlapping with the Eocene–Oligocene transition (EOT), a period of global cooling, before the orogenesis of the Andes. b) Gene reconciliation tree topology (Astral-III) with branch lengths corresponding to the coalescent units (CUs). Short branches are proxy of incomplete lineage sorting (ILS). Of note are the very short branches at the base of Ithomiini species, which indicate rapid speciation, which can also be seen by the overlapping confidence intervals (CIs) in the date phylogeny (a). c) Principal component analysis (PCA) of the read counts of cOGs, showing very distinct and diverse expression profiles of the antennal transcriptomics of the three Ithomiini butterflies studied. Females and males samples are showed in different colours.

Across the 15 species selected for our analysis, we identified 5,077 single-copy BUSCO genes, which, once concatenated, resulted in an alignment of 4.1 Mb of which 1.7 Mb parsimony informative. The alignment was then used to build a species tree under the maximum likelihood (ML) framework and a gene tree reconciliation, to reconstruct a first approximation of the species tree. The two approaches returned identical topologies (Fig. 2a and b). However, while the Ithomiini outgroup shows high coalescent units (CUs), the branching within Ithomiini is characterized by very low CUs, possibly indicating a rapid diversification with a high amount of incomplete lineage sorting (ILS). Based on the phylogenetic tree and different calibration points (see Materials and Methods), we further calculated the divergence time and substitution rate. The analysis indicated that Danainae diverged between 41.7 and 70.5 million years ago (Ma) (95% CI; median 54.6 Ma), while the Ithomiini during the Oligocene, between 24.1 and 42.1 Ma (95% CI; median 32.3 Ma), both overlapping with the latest estimations for these groups (Kawahara et al. 2023). The topology of the phylogenetic tree shows some differences compared with the previously reported phylogeny of Ithomiini (Chazot et al. 2019), which was based on only nine nuclear gene fragments and a mitochondrial fragment. Both studies overlap on the temporal framework of Ithomiini (CI, 24.1 to 42.1 Mya in this study and 22.75 to 30.99 Mya in Chazot et al. 2019), and in placing *Melinaea* spp. as the first diverging branch from the stem of the tribe. The difference is in the placing of the genus *Mechanitis*, which we recover as branching later in time.

### Diversity and Independent Duplications in Chemosensory Genes Across Nymphalids

**Fig. 3.**
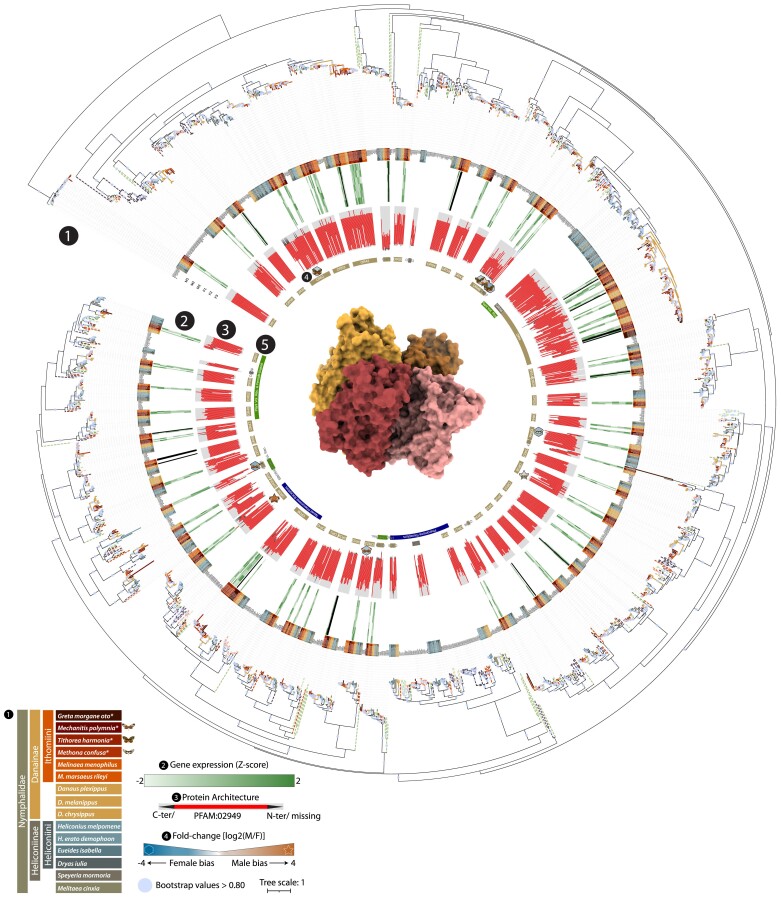
Phylogeny of odorant receptor gene family. The ML phylogeny of ORs; the circle size on branches indicates where the bootstrap is higher than 0.80 and are proportional to their values; (1) branch colors are associated to different species, while dashed line to lineages used as references, while sequences annotated in this study are highlighted in the first inner circle. In (2) the heatmap shows the expression levels in the six samples, three males (M1 to 3) and females (F1 to 3); (3) diagram showing the length of the annotated protein and the region that is occupied by the conserved domain. The arrows indicate C-terminus and/or N-terminus that are missing from the protein; (4) fold-change (log_2_ transformed) of the DEGs, also indicated by the shape (hexagon for female-biased and star for male-biased). The protein structure in the center depicts the general shape of a tetramer of a typical odorant receptor. More detailed information regarding the orthology inference, genomic locations, copy number variations within orthologous groups, and gene expression can be found in supplementary tables S2, S5, and S8, Supplementary Material online. Expression levels can also be observed in Fig. 7a.

**Fig. 4.**
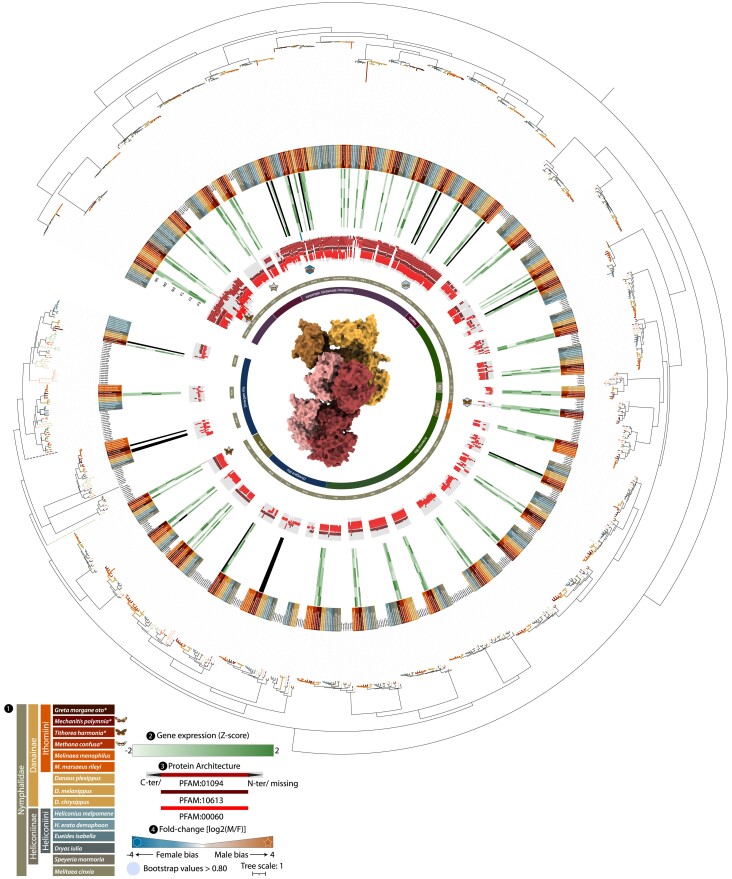
Phylogeny of ionotropic (glutammate) receptor gene family. The ML phylogeny of IRs and IGluRs, the circle size on branches indicates where the bootstrap is higher than 0.80 and are proportional to their values: (1) branch colors are associated to different species, while dashed line to lineages used as references, while sequences annotated in this study are highlighted in the first inner circle. In (2) the heatmap shows the expression levels in the six samples, three males (M1 to 3) and females (F1 to 3); (3) diagram showing the length of the annotated protein and the region that is occupied by the conserved domains. The arrows indicate C-terminus and/or N-terminus that are missing from the protein; (4) fold-change (log_2_ transformed) of the DEGs, also indicated by the shape (hexagon for female-biased and star for male-biased). The predicted/putative protein structure in the center depicts the general shape of a tetramer of a typical ionotropic receptor. More detailed information regarding the orthology inference, genomic locations, copy number variations within orthologous groups, and gene expression can be found in supplementary tables S3, S6, and S8, Supplementary Material online. Expression levels can also be observed in Fig. 7a.

**Fig. 5.**
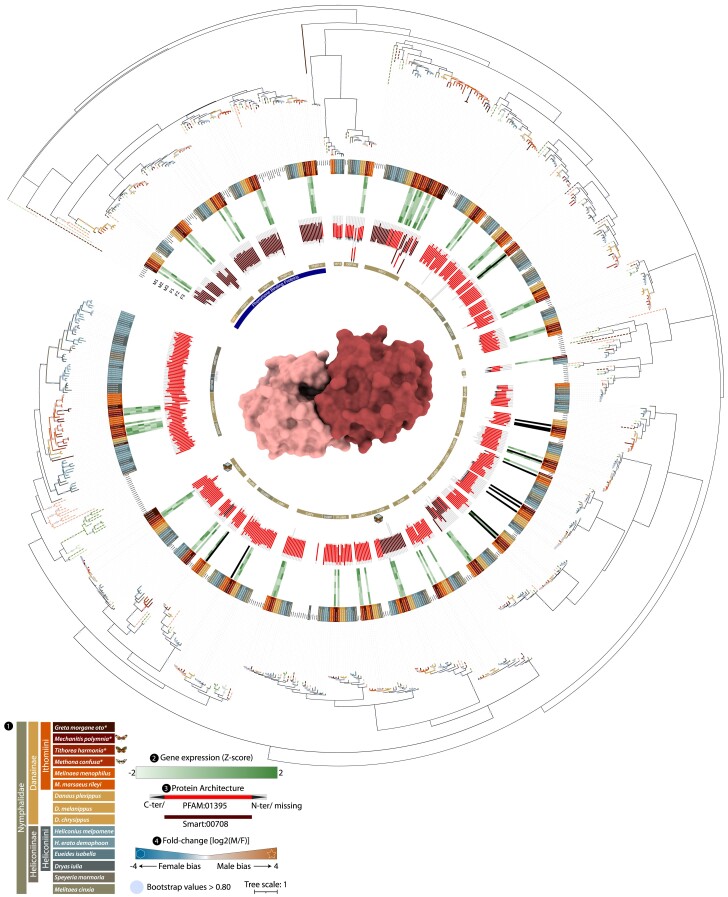
Phylogeny of odorant binding protein gene family. The maximum likelihood phylogeny of OBPs, the circle sizes on branches indicate where the bootstrap is higher than 0.80 and are proportional to their values: (1) branch colors are associated to different species, while dashed line to lineages used as references, while sequences annotated in this study are highlighted in the first inner circle. In (2) the heatmap shows with the expression levels in the six samples, three males (M1 to 3) and females (F1 to 3); (3) diagram showing the length of the annotated protein and the region that is occupied by the conserved domains. The black arrows indicate C-terminus and/or N-terminus that are missing from the protein; (4) fold-change (log_2_ transformed) of the DEGs, also indicated by the shape (hexagon for female-biased and star for male-biased). The protein structure in the center depicts the general shape of a homodimer of a typical odorant binding protein. More detailed information regarding the orthology inference, genomic locations, copy number variations within orthologous groups, and gene expression can be found in supplementary tables S4, S7, and S8, Supplementary Material online. Expression levels can also be observed in Fig. 7a.

We fully annotated our four new Ithomiini genomes using a semiautomated pipeline (see Materials and Methods), but given the particular focus of the current study, we also manually curated all antennal chemosensory receptors in all 15 species of our dataset to remove any bias caused by different annotation procedures in previously published genomes. This resulted in 65 ORs, 23 IRs, 16 IGluRs, and 35 OBPs on average per species, and a total of 2,113 annotated chemosensory genes (Figs. 2 to 5; supplementary table S2 to S7, Supplementary Material online). Not all loci show complete functional domains. Among all chemosensory gene families, ORs show the largest turnover rate and diversification. Overall, looking at loci with complete domains, Ithomiini have on average 67 ORs per species, the same as for Heliconiinae, and in line with their estimated olfactory glomeruli number of related ithomiines (Montgomery and Ott 2015; Morris et al. 2021). The complete phylogenetic tree of ORs resulted in 49 orthologous groups (OGs). One of these OGs, containing OR4 shows a major expansion within nymphalids, with a total of 107 genes across the 15 species. Of note, we found an Ithomiini-specific loss of one of the known pheromone receptor clades (OR2, OR5, OR13). In fact, while *Danaus* species have genes within OR2 and OR13, OR5 seems to be present only in Heliconiinae. This loss could be balanced by independent expansions of the other pheromone receptor clade ORs (OR30 and OR38) within Ithomiini, named “Novel” by Bastin-Héline et al. (2019). Specifically, for OR38, in-paralogs are present in the Heliconiini, *Dryas iulia* (three copies), in *Danaus plexippus* (two copies), and in the Ithomiini, *Melinaea menophilus* (two copies), *G. morgane* (four copies), *M. confusa* (four copies), and *T. harmonia* (six copies). In contrast, for OR30, there is an expansion in the Heliconiini *Heliconius* species (two copies) and an extra copy in *Heliconius erato* (three copies, in total), *D. plexippus* (three copies), and in the Ithomiini *M. polymnia* (two copies) and in the *Melinaea* species (two copies). Independent and repeated duplications of OR51 are present in all nymphalids. The OG duplicated three times at the base of Heliconiinae, followed by other species-specific duplication events, including an independent duplication at the stem of Danainae. Similarly, OR46 was duplicated at the stem of Danainae, with a further duplication in *G. morgane* (three copies located on the same scaffold). OR56, which seems to be associated with detection of plant volatile compounds (Bastin-Héline et al. 2019), seems to be not only specific to Heliconiinae but expanded at least three times. Finally, there are two major expansions specific to Ithomiini, one within OR53, which has generally two copies in nymphalids, further expanded in *Melinaea* spp. (three or four copies), *M. confusa* (four copies), and *G. morgane* (three copies), and one within OR42, which shows multiple duplications in Danainae, with several other independent expansions within Ithomiini, resulting in between four and six copies.

Compared with ORs, IGluRs and IRs show a much more conserved pattern. In total, we identified 41 OGs, with 15 IGluR OGs and 26 IRs. Within IGluRs, we ascribe two ionotropic co-receptors (CoIR), CoIR8 and CoIR25, as they cluster within all the IGluRs. Within Ithomiini, almost all IGluRs are present in single copy, although for four receptors, six more short fragments were also identified in *G. morgane* and *M. confusa*. Among the other IRs the Lepidoptera-specific (LS-IRs) and divergent IRs show the highest turnover. IR1b seems to be present only in Ithomiini and was lost in the rest of nymphalids, and duplicated in *Melinaea* ssp. Within the divergent IRs, IR7d4 seems to be lost in all ithomiines, while IR7d2, IR7d4, and IR143 underwent duplications in different ithomiine species.

OBPs show a strong pattern of conservation (Fig. 5), with very few exceptions within the antennal binding proteins (ABPs). These exceptions include ABP8, which shows multiple duplications within *G. morgane*, with four copies, while ABP1 shows numerous independent duplications in Heliconiini, *Melinaea* spp., and Ithomiini. Specifically, a total of three copies were identified for *M. confusa* and *M. polymnia* and five and six for *T. harmonia* and *G. morgane*, respectively. Notably, the OG of ABP6 shows huge expansion in all included Lepidoptera. After a first duplication in Heliconiini, the gene was duplicated several other times, with some losses, resulting in between 11 and 14 copies per species within Heliconiini. Within Ithomiini, ABP6 independently duplicated in different lineages, resulting in between three and five copies of the gene per species (see Fig. 5 and supplementary table S7, Supplementary Material online for more details).

### Interspecific and Intraspecific Diversity in Antennal Gene Expression

To understand sexual dimorphism at the level of antennal gene expression, we analyzed transcription expression level in both sexes of three species, *M. confusa*, *T. harmonia*, and *M. polymnia* (three biological replicates per sex; see Materials and Methods). A total of 686 million reads were obtained after sequencing all eighteen libraries. All libraries show good statistics in terms of GC distribution, quality of sequences, and redundancy (supplementary figs. S6 to S8, Supplementary Material online). On average 38 M pair-reads were obtained for each sample, which resulted in a 33 M uniquely mapped reads on average (87%). Although one sample (*M. confusa* F1) shows a lower degree of percentage of uniquely mapped reads, the absolute values (22 M) and PCA (supplementary fig. S6, Supplementary Material online) show no bias or possible artifacts and similar degree of variance compared with the other species. Overall, females of *T. harmonia* and *M. polymnia* showed more variance compared with *M. confusa*, where instead males showed more diversity. Considering a minimum threshold of 10 transcripts per million (TPM) on average, the three species expressed similar numbers of genes (on average ∼6.4k genes per species).

To explore expression profiles across species, we used single-copy positional OGs (scOGs; see Materials and Methods) and clustered counts on a PCA. This shows a strong pattern of interspecific divergence in expression patterns (Fig. 2c), which is also evident from very long branches in the tree obtained by clustering samples by gene expression (supplementary fig. S9, Supplementary Material online). The topology shows *M. confusa* and *T. harmonia* forming sister clades, as in the phylogenetic tree (Fig. 2), showing that phylogenetic signal can be detected in gene expression regardless of the short branches at the base of Ithomiini. Overall male transcriptomes seem to be more similar compared to females, which again, show higher interspecific heterogeneity.

**Fig. 6.**
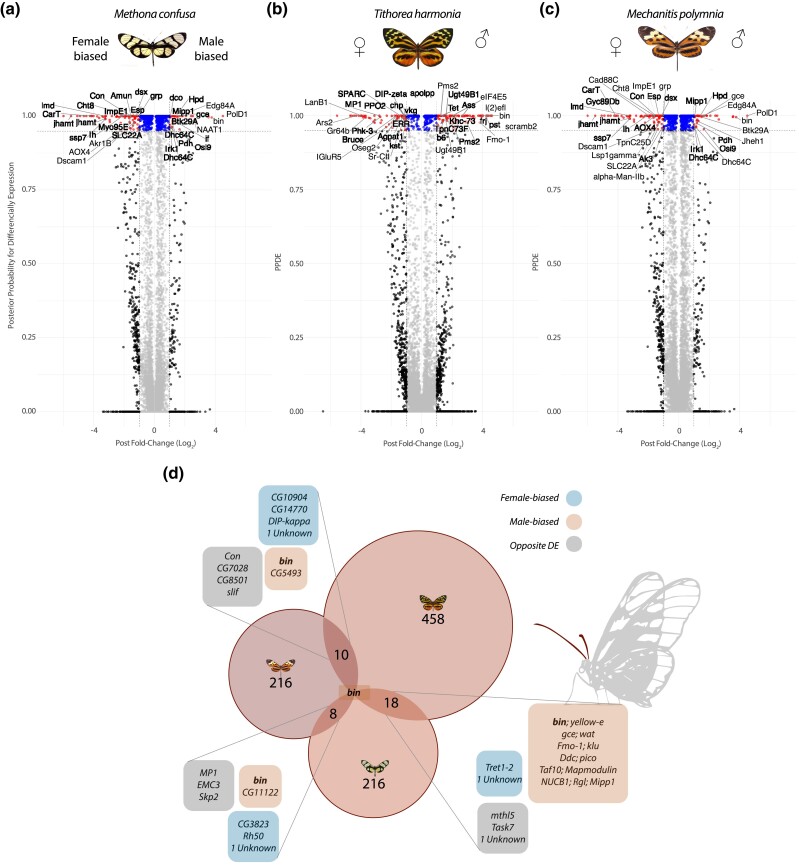
Antennal differential gene expression in the three species of Ithomiini. a to c) Volcano plots showing the relation between fold-change (FC) (log_2_ transformed) and their Bayes factors (BFs) of the antennal gene expression. Above the horizontal dased line there are the significantly differential expressed genes (DEGs; posterior probability > 0.95). Between the vertical dashed lines genes with a fold-change (FC) below |1|; while externally genes with a FC > |1|. For each plot, female-biased and male-biased genes are on the left and right, respectively, for *M. confusa*, *M. polymnia*, and *T. harmonia*. d) Venn diagram showing the DEGs in common between species. The numbers in the main circles are the scOGs used for the comparisons. For each comparison, genes are grouped in boxes according to the concordance of expression (colored boxes) or not (gray boxes).

To characterize sex-specific genes in the antennal transcriptomes, we identified 499, 380, and 772 differentially expresses genes (DEGs) between sexes in *M. confusa*, *M. polymnia*, and *T. harmonia,* respectively (Fig. 6; supplementary fig. S10 and table S8, Supplementary Material online; posterior probability > 0.95). Turning to species-specific patterns:

- Within *T. harmonia*, we found significant terms only in male-biased genes (upregulated genes in males; adjusted *P* < 0.05). The 30 GO terms involve 362 of the 426 DEGs enriching almost exclusively biological processes involved in the biosynthesis of lipids, monocarboxylic acids, fatty acids, terpenoids, organonitrogens, their regulation, and response to external stimuli (supplementary table S9, Supplementary Material online). Among the 346 female-biased DEGs are *doublesex* (*dsx*), which controls somatic sexual differentiation and courtship behavior and mediates the development of sex-specific pheromone organs in butterflies (Prakash 2020); *juvenile hormone acid methyltransferase* (*jhamat*), which has effects on courtship behavior in *Drosophila* (Wijesekera et al. 2016); *SLC22A*, a cation transporter implicated in the regulation of olfactory learning (Gai et al. 2016); and *Epidermal stripes and patches* (*Esp*), which encodes a protein involved in female remating receptivity (Findlay et al. 2014).
- Within *M. polymnia*, there are 221 and 159 male-biased and female-biased genes, respectively. The male-biased genes include *farjavit* (*frj*), a lysophospholipid acyltransferase, *Dpr-interacting protein γ*, *Kinesin heavy chain 73* (*Khc-73*), and *bric a brac* (*bab*); while in female-biased genes appear *apolipophorin* (*apolpp*) and *Dpr-interacting protein ζ* (*DIP-κ*), all of which related to lipid processes.
- Within *M. confusa*, we detect a very skewed proportion of male-biased versus female-biased DEGs with 85 and 414, respectively. Male-biased DEGs involve seven members of the cuticular proteins (*Cpr*) which are highly expressed (Log_2_ FC > 4.4); but also, *ebony* and *yellow-e* involved in dark pigmentation in butterflies (Zhang et al. 2017); T*rpA1*, a thermotactic sensor; *defective proboscis extension response 18* (*dpr18*), and *pumilio* (*pum*) involved in synapse organization and long-term memory (Dubnau et al. 2003). In the female-biased DEGs, we find *sarah* (*sra*), which in *Drosophila* is involved in the regulation of female receptivity, post-mating receptivity (Ejima et al. 2004); *murashka* (*mura*) implicated in long-term memory (Akalal et al. 2011); and *supernumerary limbs* (*slmb*), involved in the regulation of circadian clocks (Srikanta and Cermakian 2021).

Finally, turning away from species-specific patterns, to test for a deeper phylogenetic expression pattern between sexes, we assessed the amount of overlap in sex-biased gene expression between species using single-copy OGs. Echoing the results from the PCA, we find very little overlap between DEGs among the three species (18, 10, and 8 for *T. harmonia* vs. *M. confusa, T. harmonia* vs. *M. polymnia*, and *M. confusa* vs. *M. polymnia*, respectively, Fig. 6). Between *M. confusa* and *T. harmonia*, there are 18 DEGs in common, three of these have opposite biases, while 15 show the same bias (upregulated only in males or females), of these 13 are male-biased. Among these are *germ cell-expressed bHLH-PAS* (*gce*) and *klumpfuss* (*klu*); while among the shared female-biased genes, there is trehalose transporter, a mediator in the bidirectional transfer of trehalose and regulating trehalose levels in the hemolymph; and between *T. harmonia* and *M. polymnia*, there are 10 DEGs in common, 4 with opposite gene expressions, 4 unidirectionally expressed in females, and 2 in males. Between *M. confusa* and *M. polymnia*, there are the least DEGs in common, only eight, three and two unidirectionally expressed in females and two in males, respectively, and three discordant. There is only one gene shared in all comparisons: *biniou* (*bin*), which was always found as male-biased with a very high fold-change (Log_2_ FC > 5).

### Sex-Biased Antennal Chemosensory Gene Expression Patterns

We detected high transcriptomic diversity for chemosensory genes highlighting deep transcriptomic differences that are likely the result of adaptations to recognize odorant volatiles during mate choice and host plant selection. Except for only 19 ORs, all the remaining 187 (91%) ORs are expressed across the 3 species, with a higher proportion of the OR gene family being expressed compared with IRs + IGluRs or OBPs, of which 101 (85%) and 83 (86%) are expressed, respectively. The OGs OR68, IR143, IR7d4, OBP15, and OBP32 are consistently not expressed in all the three species. Also, five duplicated ORs/IRs from *M. confusa* (OR42_Loc44_, OR4_Loc22_, IGluRIa _Loc46_, IGluR_Loc45_, IR75pA _Loc47_), three from *T. harmonia* (OR24_Loc40_, OR4_Loc32_, OR247_Loc30_), and four from *M. polymnia* (OR24_Loc11_, OR4_Loc12_, OR4_Loc13_, OR38_Loc29_) are not expressed and are therefore likely nonfunctional, expressed in the larval stage, or expressed in other non-antennal tissues.

To explore sex-specific chemoreception, we identified OR genes which display sex-biased DE in the analysis described above. Among all the 201 ORs tested, 10 show DE between sexes. Specifically, *M. confusa* has one female-biased (MpolOR44_Loc34_) and one male-biased (MpolOR8_Loc11_) receptor. The male-biased gene belongs to the same OG that is also male-biased in another butterfly, *Heliconius cydno* (van Schooten et al. 2020), while the female-biased gene belongs to an OG where no functional information is available, therefore showing the association between this OG and sex for the first time. In *T. harmonia*, we detected four DE ORs, all female-biased. Two of these genes belong to the same OG37 (TharOR37_Loc31_, TharOR37_Loc33_), and are located on the same scaffold, 35 kbp apart on opposite strands. This OG is also lost from almost all species from our dataset, and an intact functional domain is present only in three Ithomiini species (*T. harmonia, M. menophilus*, and *Melinaea marsaeus rileyi*) (see the tree topology in Fig. 3). The other two female-biased genes (TharOR46_Loc08_ and TharOR51_Loc57_) belong to OGs that have been found to be sex-biased in *H. cydno* (van Schooten et al. 2020); TharOR46_Loc08_ with the same female-bias and TharOR51_Loc57_ being male-biased in *Heliconius*. TharOR46_Loc08_ was also found to be sex-biased in *M. polymnia*, although in the opposite direction (MpolOR46_Loc43_, female-biased), together with the female-biased MpolOR40_Loc43_. *M. polymni*a has two other DE ORs, both male-biased: MpolOR23_Loc31_, also found to be male-biased in *Spodoptera litura* (Feng et al. 2015), and MpolOR30_Loc23_, which belong to a novel pheromone receptor clade (Walker III et al. 2019). Only one OR, OR46, is sex-biased across all species, but not in a consistent direction.

Among all the 86 IRs and IGluRs tested, 3 IRs and 3 IGluRs display DE between sexes. In *M. confusa*, the coreceptor CoIR8 was detected as male-biased and the GluRIIb female-biased. In *T. harmonia*, the “Lepidoptera-specific” IR87 and the antennal IR31 are male-biased and female-biased, respectively. While the IGluR1 is identified as male-biased, *M. polymnia* instead shows a single female-biased receptor, the IGluRIa, with a very high fold-change (FC_log2_ = 3.9). Finally, although OBPs constitute the higher fraction of ACGs, none of the “canonical” pheromone binding proteins (PhBPs) shows differential expression between the sexes, but two other transcripts do, both in *T. harmonia*: the ABP8 and ABPX, both of which are female-biased. Notably, the functional domain of the ABPX in the Ithomiini, instead of showing the canonical conserved domain of other ABPs and GOBPs (PFAM:01395), as observed for this OG in Heliconiinae, similar to the functional domains of the PhBPs (smart00708). This shift seems to also occur within the orthologs of ABP1 and OBP29, possibly hinting at a gain-of-function.

### Candidate ORs Linked to the MGC in Ithomiini Butterflies and High Expression of Coreceptors

**Fig. 7.**
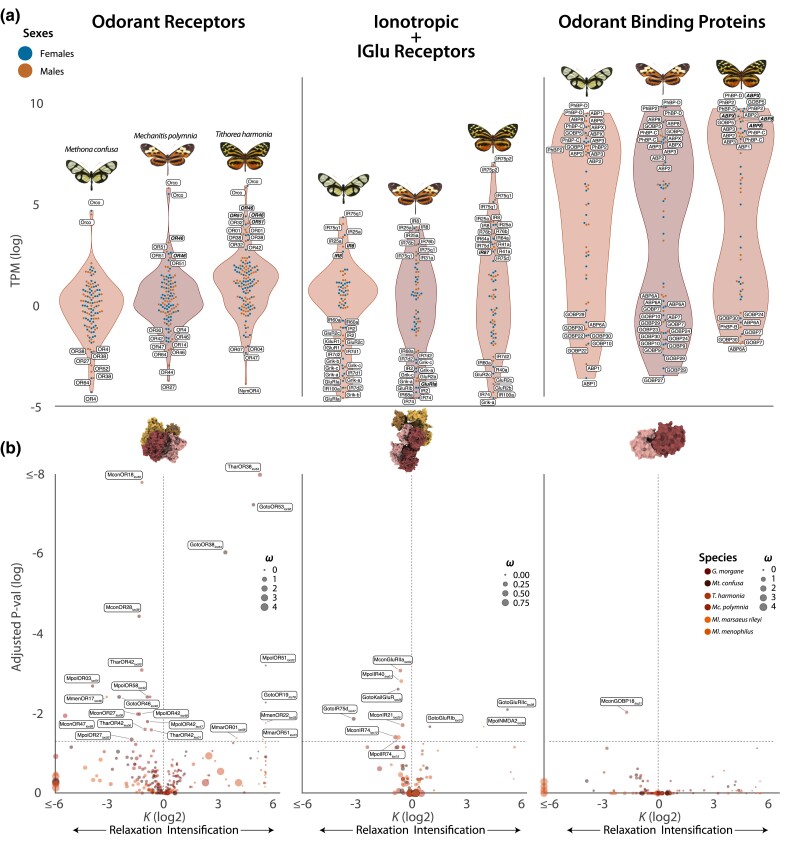
Chemosensory gene expression and the distribution of selective shift. For each chemosensory gene family, we show the distribution of their expression level (TPM) in each of the three genes (upper half) and the distribution of *k*, the relaxation/intensification index for each coding sequence in all the six Ithomiini species included in this study (bottom section). The horizontal dashed lines indicate the bottom threshold level (adjusted *P* < 0.05), while the vertical one the shift between the relaxation (log_2_(k) < 0 and intensification (log_2_(k) > 0). Species are color coded, while their size is relative to the *d*_N_/*d*_S_ (*ω*) for that particular locus.

A relatively large proportion of total antennal gene expression is accounted for by ACGs, ORs, IRs, and OBPs. On average, >5% of expressed transcripts belong to these genes (*M. confusa*, 5.9%; *T. harmonia*, 5.9%; *M. polymnia*, 5.2%). Females of *M. confusa* and *T. harmonia* express more ACGs compared to males (∼7% of fold-enrichment), while in *M. polymnia* males express more ACGs (supplementary table S8, Supplementary Material online). Notably, OBPs constitute over the 96% of those transcripts (Wilcoxon rank-sum test “one-sided” *P* < 2.2 × 10^−16^) (Fig. 7). The shape of the distribution of gene expression differs between gene families, with ORs having the smallest variance and OBPs the largest. OBPs also have a bimodal distribution of expression (Hartigans’ dip test *D* = 0.050155, *P* value < 0.005) (Fig. 7). Notably, the distributions of OR gene expression varies among species, with *M. confusa* having lower expression of ORs than *M. polymnia* (medians: 1 TPM and 1.3 TPM; Wilcoxon rank-sum test “one-sided” *P* = 0.017), and *M. polymnia* lower than *T. harmonia* (median: 2.9 TPM; Wilcoxon rank-sum test “one-sided” *P* = 4.35 × 10^−6^) (Fig. 7). In contrast, IRs and OBPs do not show this significant interspecific variation.

To putatively attribute the expanded MGC glomeruli to ORs, we also looked at the within-ACG expression in our species. In *M. confusa*, ORs seem to show more restricted variation in gene expression of ORs, with only Orco being markedly more expressed (Fig. 7). This is consistent with the monomorphic nature of their antennal lobe glomeruli (Fig. 1e), and the absence of macroglomeruli in this species (Morris et al. 2021). In the other two species, *M. polymnia* and *T. harmonia*, the distribution of ACG expression has a longer tail (Fig. 7). In *M. polymnia*, alongside the c-receptor Orco, there are two more highly expressed transcripts OR46 and OR51, which show sex-biased expression. In *T. harmonia*, excluding Orco, there are five highly expressed receptors, two which are also found in *M. polymnia* (OR46 and OR51), and three more: OR32, OR42, and OR38, which belong to the “novel” pheromone clade (Figs. 2 and 7). Within IRs and IGluRs, the coreceptors CoIR8 and CoIR25a and the antennal IR75q1 are highly expressed in all the species. In the upper tail of the distributions of the other two species, we find the coreceptors CoIR76, while in *T. harmonia*, the most expressed IRs is the antennal IR75pB, which is three times more expressed than Orco (FC_log2_ > 1.64). OBPs are most abundant component of the ACGs by far. Within them, the ABPs and the PhBPs are the most dominant transcripts. Looking across all three species, the most abundant OBPs are always the PhBPs (PhBP-C, PhBP-D, PhBP2), the ABPs (ABP2, ABP3, ABP8 and ABPX), and the GOBP5, while the bottom part of the OBP expression distribution is always occupied almost exclusively by GOBPs with ABP6A and PhBP-B in *T. harmonia* (Fig. 7).

### Differential Selection Regimes Across Chemosensory Genes

To identify differential selection regimes across Ithomiini, we performed the RELAX test computing the *k* parameter on each annotated ORs, IRs, and OBPs across all Ithomiini species. Comparisons of the *k* distributions also provide a proxy to understand which of the ACG families is the most and lesser heterogeneity within Ithomiini. The scan for relaxation/intensification of positive/purifying selection shows diverse patterns among chemosensory gene families. ORs are by far the most dynamic with a median *k* of 1 but with a standard variation of 13.5. In comparison, IRs plus IGluRs and OBPs, have a median still around 1, but with a standard variation of 7.9 and 11.3, respectively. Within ORs we found 8 loci to be under intensified selection and twice as many and 16 to be under relaxed selection; showing strong selection turnover is acting on ORs. Five of the relaxed loci are at the bottom distribution of OR expression, hinting to a possible loss of function. Among the eight loci under intensification, MpolOR51_Loc22_ and TharOR38_Loc64_ are among the most highly expressed loci, with OR38 belonging to the “novel” pheromone clade. Furthermore, the loci of OR38 and OR51 are also under intensification in *G. morgane* and *M. marsaeus rileyi*. In fact, there is a sign of intensification in two ORs of *M. marsaeus rileyi* (MmarOR51_Loc31_ and MmarOR01_Loc51_) and one in *M. menophilus* (MmenOR22_Loc03_), two closely related species. Interestingly, ORs that show a high rate of relaxation include five genes derived from OR42, a highly duplicated OG (Figs. 3 and 7) (e.g. TharOR42_Loc03_ and MpolOR42_Loc48_), supporting the inferred pattern of duplication with pseudogenization. Within IRs and IGluRs, there are nine loci under relaxed selection, among them MpolIR40a_Loc01,_ which is lowly expressed, and two that belong to the OG OR74, supporting a general trend toward the loss of these genes within nymphalids. Only three of these receptors are identified as evolving under intensified selection. The loci under intensification are not the IRs but the IGluRs, two in *G. morgane* (GotoIGluRIb_Loc01_ and GotoIGluRIIc_Loc27_) and one in *M. polymnia* (MpolNMDA_Loc38_), genes that may play a role in synaptic plasticity, synaptogenesis, excitotoxicity, memory acquisition, and learning. Finally, consistent with their general conservation, OBPs show the least variation in selection regime. The lack of selection turnover is an indication of how important these genes are for the correct functionality of the whole chemosensory circuit.

## Discussion

The ability to detect odors plays a fundamental role in organisms, not only because it enables them to recognize environmental chemical signals, but also because it allows communication between individuals. This is particularly true in Ithomiini butterflies where the detection of specific compounds is linked to strong inter- and intra-sexual behaviors. Therefore, chemosensory organs should harbor a complex pattern of gene expression not only strictly related to chemosensory genes, but a variety of non-chemosensory genes that have a great importance to support neuronal sensory function cells and to regulate the stimuli (Schmidt and Benton 2020; Scalzotto et al. 2022). It is therefore plausible that selection could act in modulating the expression of both chemosensory and non-chemosensory genes generating distinct expression patterns within and between species.

In this study, we generated high-quality/highly contiguous reference genomes for four Ithomiini species, a tribe of diurnal butterflies with reliance on olfactory cues, at least partially reflecting major phylogenetic and ecological differences across the tribe. Using antennal transcriptomics, we aimed to assesses divergent patterns of chemosensory evolution, and to putatively associate expression profiles with neuroanatomical differences (i.e. the presence/absence of MCGs) between species. Specifically, based on patterns of neuromorphological variation between ithomiines and other butterflies, and within ithomiines we predicted (i) a molecular signature that parallels the expansion of specific glomeruli in the ithomiine antennal lobe (i.e. particularly high expression of a small number of ORs); (ii) evidence of sexual dimorphism in some, but not all, of these genes; and (iii) interspecific differences, with *Methona* displaying reduced variance and dimorphism in chemosensory gene expression compared to other ithomiines, in line with the homogeneous structure of this species’ antennal lobe, which is more typical of other butterflies (Morris et al. 2021).

To address the first two points, we examined at the expression level of ACGs, exploring two patterns: the relative expression within ACGs and the differential ACG expression between sexes. From a behavioral perspective, the presence of sex-specific receptors could provide insights into the genes that hold greater relevance for each sex. We can speculate that, although in females there may be selection for an ability to choose males with high PA concentrations in their nuptial gifts, and for locating and choosing suitable host plants for egg deposition, in males there is a unique and strong selective pressure toward sensing pheromones, ultimately due to male-to-male competitive interactions, which then drive the evolution of neuroanatomical emergence of the MGCs (Morris et al. 2021). Because each glomerulus in the antennal lobe is associated with OSNs expressing one or two chemosensory receptors, and because MGCs in moth s are typically involved in processing olfactory pheromones, we might expect to see skewed distributions of chemosensory receptor expression, with ORs more associated with MGCs being the ones that are more highly expressed. Among the three species *M. confusa*, a species, which lacks MGCs (Morris et al. 2021), showed the least differentiation both in terms of sex-specific ACGs, with one male-biased and one female-biased OR, and the least skewed distribution of ACGs. In *M. polymnia*, a species with MGCs, we found four sex-biased ORs, these correspond to two female-biased ORs: OR23 and OR40, the latter being orthologous to SlitOR40, associated with the reception of plant volatiles (Revadi et al. 2021); and two male-biased ORs: OR46 and OR30, the first being the most expressed OR (excluding the ubiquitous co-receptor Orco), and the second belonging to the “novel” pheromone receptor clade, with possible implications for convergent evolution of reliance on long-distance pheromone detection with moths. Finally, *T. harmonia* showed the most skewed distribution pattern, with six highly expressed ORs, two of which are female-biased. They also show high expression of several other IRs, one of which is male-biased, and OBPs, two of which are female-biased. While extensive data on the antennal lobe morphology of *T. harmonia* is currently lacking (but see Fig. 1), the presence of sexual dimorphism in hind-wing hair pencils, which are present only in males (Fox 1940), in common with other ithomiini that show enlarged glomeruli, would suggest that these outlying ORs (OR46 and OR51) are associated with MGC glomeruli.

In terms of interspecific differences among species, we also show how the antennal transcriptome is profoundly divergent between related species, suggesting distinct adaptations in sensing their environment, and possibly hostplants (Fig. 2c, 7). The five genera of Ithomiini presented in this study reflect the hostplant diversity for the tribe, with *Melinaea* using *Solandra* spp. as hostplants, *Methona* using *Brunfelsia* spp., *Tithorea* using Apocynaceae, *Greta* using *Cestrum* spp., and *Mechanitis* uses *Solanum* spp. (Willmott and Freitas 2006). Males are also known to be attracted to various plants containing PAs (Pliske 1974a; Brown 1985). Volatile “esterifying acids” liberated from alkaloids in rotting plant tissue provide olfactory cues for locating these plants, and the “hairpencil” glands of males in certain genera contain a lactone structurally similar to the attractive acids (Schulz 1998). The release of these compounds appears to act as a male territorial-recognition pheromone and allomone, repelling not only conspecific males but also those of other lactone-producing species (Pliske 1975b). The lactones have further significance in allowing males to terminate male-to-male intra- and interspecific courtship pursuits. Male hairpencil components showed a great diversity of compounds across ithomiines (Schulz et al. 2004), and among the species sampled here, *M. polymnia* showed the greatest diversity of PAs, with four different identified classes of compound, followed by *Tithorea* and *Melinaea*; *G. morgane* with PAs and lactones, while *Methona* extracts contained no PA nor lactones, mirroring their less specialized antennal lobe morphologies. Therefore, this huge diversity in hostplant preference and chemical communication could be correlated with the molecular divergence we observed across these species.

Similar patterns of diversity in antennal transcriptomes have previously been found in other lepidopteran radiations, including *Heliconius* species, where the antennal transcriptomics shows the largest diversity of expression patterns compared with mouthparts and legs (Wu et al. 2022). Similarly, in the only other ithomiine study on antennal transcriptomics, in two subspecies of *M. marsaeus*, Prunier et al. (2021) found twice as many DE transcripts in the antennae (1,028 transcripts), compared with imaginal disks. In our study, the species-specific selective pressures driving the evolution of gene family composition and gene expression patterns seem to be so profound that the number of DEGs shared between sexes is minimal across species. The small number of shared genes within these DEGs do include *Gce*, a male-biased gene in *Drosophila,* found to bind to the juvenile hormone, which has been shown to determine sex dimorphisms in the gut regulating intestinal stem cell proliferation (Millington and Rideout 2020); *Klu*, a transcription factor involved in determination of the identities of neuroblast lineages in the central nervous system (Xiao et al. 2012); and *biniou* (*bin*), which is always male-biased across the three species, which encodes for a transcription factor with important regulator functions for the development of the visceral musculature of the midgut (Zaffran et al. 2001), and which is also expressed uniquely in *Drosophila* males (Brown et al. 2014). Together, these represent candidates for the exploration of conserved network gene expression in sex dimorphism in butterflies.

Looking at within species sexual dimorphism at the transcriptomic level for non-chemosensory genes, we found a recurrent signal from genes related to lipid processes. In *T. harmonia*, we found 66 DEGs that almost exclusively enrich biological processes involved in the biosynthesis lipids and hormone metabolic process, while within *M. Polymnia*, there are male-biased genes, such as *farjavit* (*frj*), a lysophospholipid acyltransferase, which is also related to synaptic transmission, and the genes *scramblase*, *Dpr-interacting protein γ*, and *Kinesin heavy chain 73* (*Khc-73*), which may all be implicated in regulation of olfactory learning (Guven-ozkan et al. 2020). *Bric a brac* (*bab*) is also highlighted and has been shown to control male sex pheromone choice in the moth, *Ostrinia nubilalis* (Unbehend et al. 2021). Similarly, in the female-biased genes, *apolipophorin* (*apolpp*), a lipid transporter (Ugrankar et al. 2019), and *Dpr-interacting protein ζ* (*DIP-κ*), involved in establishment of synaptic specificity at neuromuscular junction (Bornstein et al. 2021), are both upregulated. Although the role of lipid composition in OR signaling is unclear, genetic studies in *Drosophila melanogaster* have revealed that lipid transporters, such as ATP8B, have pivotal roles in olfactory sensory neurons classes related to pheromone ORs (Liu et al. 2014; Soo et al. 2014). These transporters are required to flip aminophospholipids (e.g. phosphatidylserine) between membrane leaflets, potentially affecting the morphology of the ciliated dendrites, which in turn could play a crucial role in facilitating the interaction between odor molecules and the OSN, constantly refreshing the OSN surface, ensuring that odor molecules can effectively reach and interact with the receptors (Garcia III et al. 2018; Schmidt and Benton 2020).

The comprehensive investigation of ACGs across six representative species of the Ithomiini tribe has yielded a wealth of insights to suggest a link between the genetic diversity, ecological specialization, and sensory adaptation of these species. The multifaceted landscape of chemosensory gene evolution, expression, and potential functional significance offers a rich platform for understanding the genetic underpinnings of sensory perception and its role in driving behaviors and ecological interactions. In our study, ACGs for 15 species were manually curated and, compared to a previous study on ACGs in *M. marsaeus* and *M. menophilus* (Prunier et al. 2021; Gauthier et al. 2023), we found a slightly different number of ACGs. This difference likely reflects our wider phylogenetic framework allowing us to observe a more accurate picture of chemosensory evolution and more precisely contextualize changes that might have occurred within the Ithomiini tribe compared to other nymphalid species. Strong differences were observed within and among ACGs, such as the dramatic difference between ORs and the other ACGs (IGluRs, IRs, and OBPs) which showed significantly higher turnover and heterogeneity of selection signal. Among all IRs, the LS-IRs and the so-called divergent IRs showed the highest turnover. We detected an Ithomiini-specific clade, the IR1b, and several duplications in IR7d2, IR7d4, and IR143. In contrast, the general conservation of the OBPs within Nymphalidae, with very few exceptions such as the expansions of ABP6a, hugely expanded in Nymphalidae, suggests strong functional relevance of these genes. Odorant receptors, on the other hand, have the highest turnover rate of losses and gains, and selective heterogeneity, such as the loss of genes from the “known” butterfly pheromone receptor clade, and the expansion within the “Novel” pheromone receptor clade (OR30 and OR83), associated with moths (Bastin-Héline et al. 2019). This suggests that ORs might be strongly related to mate or host plant differentiation. Indeed, two Ithomiini-specific expansions within OR53 and OR42, orthologous to HarmOR40, are potential candidates for ORs involved in mate preference due to their affinity with terpenes (Guo et al. 2021), a class of compounds found in the androconia of the ithomiine butterfly *Ithomia salapia*, which they likely sequester from their hostplant (Mann et al. 2020).

Finally, to gain insight into selection pressures shaping the evolution of chemosensory genes, we also explored differences at the nucleotide level, identifying in ORs the gene family with the most dynamic range of shifts, and the highest number of genes under intensification. Of these, two, OR51 in *Mc. polymnia* and OR38 in *T. harmonia*, are under intensification of selection and more highly expressed compared to the other ORs, suggesting a fundamental role of these genes in these species. The diversification pattern in ORs is also evident looking at the two closely related species: *M. marsaeus rileyi* and *M. menophilus*, diverged only ∼5 Mya. They show three ORs under intensification, with possible implications in assortative mating. Overall, the variation in gene counts, expression patterns, and relaxation/intensification of selection indicate possible pivotal roles of ORs in sensory perception and behavioral responses, potentially linked to ecological adaptation and facilitating speciation. In contrast, the conserved patterns in IGluRs and OBPs suggest a more profound constraint, perhaps related to roles in maintaining core sensory functions, with a more delicate balance between gene innovation and conservation in shaping the chemosensory landscape in butterflies.

In conclusion, while in moths, expanded glomeruli in the antennal lobe, typically sexually dimorphic and responsive to pheromones, are quite common (Rospars and Hildebrand 1992; Christensen and Hildebrand 2002), in butterflies these anatomical modifications were lost and independently evolved in the hyper-diverse Ithomiini, the only tribe of butterflies currently known to possess this kind of olfactory specialization (Montgomery and Ott 2015; Morris et al. 2021). Here, by assembling the genomes from four representatives of the tribe and sequencing sex-specific antennal transcriptomics of four of them, we provide evidence that the convergent evolution of these neuroanatomical features does not involve olfactory innovations in Ithomiini, but on the other hand, they adapted already available receptors, possibly achieving the same sensory function. We show that *M. confusa*, which lacks MGC and sexual dimorphism in the antennal lobe, mirrors this difference at the molecular level, showing an absence of unusually highly expressed ORs and less sexually dimorphic gene expression compared to the other species studied here. By extension, while functional validation is obviously critical to confirming the ligands of any OR, the highly expressed ORs we identify in *Tithorea* and *Mechanitis* are strong candidates for newly described pheromone receptors in ithomiines.

In summary, this study provides a comprehensive exploration of chemosensory gene evolution, expression patterns, and potential functional implications within the Ithomiini tribe. The identification of sexual dimorphism, the presence of MGCs, and the detection of differential selection regimes enrich our understanding of sensory adaptations in butterflies. By elucidating the genetic foundations of chemosensory diversity and its ecological significance, this research contributes not only to the field of sensory ecology but also lays the groundwork for future investigations into the genetic drivers of behavior, adaptation, and speciation in insects.

## Materials and Methods

### DNA and RNA Extraction and Sequencing

Samples for *M. polymnia, T. harmonia, M. confusa*, and *G. morgane* were obtained from commercial pupae supplies, derived from outbred populations (Stratford Butterfly Farm, UK; London Pupae Supplies, UK). These species represent 3 of the 10 recognized subtribes within Ithomiini: Godyridina, Mechanitina, Tithoreina, (Chazot et al. 2019), and variable microhabitat preferences that separate mimicry complexes across open and closed canopy forest (Elias et al. 2008; Hill 2010), and antennal lobe morphologies (Morris et al. 2021). High-quality, high-molecular-weight genomic DNA was extracted as in (Cicconardi et al. 2023); 100 mg of tissue were dissected, snap-frozen in liquid nitrogen and homogenized in 9.2 mL buffer G2 (Qiagen Midi Prep Kit) adding 19 µL of RNAseA, adding 0.2 µL of protease K and incubated at ∼50 °C for 2 h. Samples were processed with a Qiagen Midi Prep Kit (Qiagen, Valencia, CA) following the manufacturer's instructions, and precipitated using 2 mL 70% EtOH and dissolved in water. To generate whole-genome sequencing data, the 10 × Chromium Library Prep was adopted alongside Illumina sequencing using 150 bp paired-end reads with NovaSeq FC S2, generating ∼40 Gbp per species, performed at the Institute of Applied Genomics (IGA), Udine, Italy.

For RNA extractions, pupae were allowed to eclose at 26 °C and 80% humidity under a 12:12 day–night regime. Butterflies were then aged in these conditions for 4 to 6 days in 1.5 m × 1.5 m × 2 m cages and fed on a 30% sugar solution. The cages included plants, *Cestrum nocturnum* and *Solanum crispum*, from the Solanacea as natural stimuli, and cuttings of *Heliotropium* (a PA source for adults). Surviving butterflies were then flash frozen in liquid nitrogen and stored at −80 °C. To capture sex-specific expression of antennal olfactory receptors, pairs of antennae were homogenized with RLT buffer by repeated aspiration with a 21-gauge needle, and RNA was extracted using a Qiagen RNeasy kit, following the manufacturer's instructions, including treatment with Qiagen RNase-free DNase to remove any remaining DNA. To achieve sufficient concentrations, three individuals were pooled in each sample according to their estimated RNA concentrations (i.e. three different specimens per replicate), with three biological replicates per sex per species for *M. polymnia, T. harmonia,* and *M. confusa*. Sufficient samples of *G. morgane* were not available at the time of sampling. Polyadenylated Illumina RNA-seq data (125 bp × 2) was carried out by University of Liverpool Centre for Genomic, for a total of 18 samples.

### 10x Genomics Linked-Read Genome Assembly and Repeat Annotation

Sequenced Illumina paired-end reads from 10X Genomics libraries were input to the Supernova V2.1.1 assembler (10x Genomics, San Francisco, CA, USA) (Zheng et al. 2016) for de novo genome assembly. No trimming was needed as per the assembler documentation. The assembly pipeline follows (Cicconardi et al. 2023). In brief the optimal amount of reads was adopted to maximize contiguity, duplication level and completeness, based on BUSCO (Benchmarking Universal Single-Copy Orthologs; V3.1.0, Insecta_odb9) statistics (Simão et al. 2015). Subsequently, assemblies were processed with Purge Haplotigs to remove haplocontigs, and Tigmint V1.1.2 (Jackman et al. 2018) was used to correct potential assembly errors. RNA-seq data were then used for scaffolding as implemented in P_RNA_scaffolder, followed by ARCS V1.1.0 (Yeo et al. 2018).

Transposable element (TE) de novo annotation is an important step during the gene annotation, but standard approaches can be highly inaccurate when analyzing genomes from nonmodel species. This is primarily due to the frequent partial status of raw consensus sequences and the high number of unclassified repeats (Platt et al. 2016; Goubert et al. 2022; Martelossi et al. 2023; Sproul et al. 2023). To address this issue, we followed a standardized pipeline described (Osmanski et al. 2023). Briefly, in the first step the pipeline employs RepeatModeler v2.0.4 (Flynn et al. 2020) with RepeatScout v1.0.6 (Price et al. 2005) to discover TEs and generate the initial repeat library. Next, we extended consensus sequences using a combination of the Ray lab's Extract_Align and Robert Hubley's DavidExtendedConSram.pl scripts, available at https://github.com/davidaray/bioinfo_tools. The extended consensus was further classified using RepeatClassifier from the RepeatModeler package. TE-related proteins and structural features were collected with the TEcurate.sh script (https://github.com/davidaray/bioinfo_tools/blob/master/TEcurate.sh) implemented with Diamond V2.1.5.159 (Buchfink et al. 2021) on the RepeatPeps.lib libraries from RepeatMasker repository, followed by TE + Aid v.0-dev (Goubert et al. 2022). The resulting libraries, one for each species, were then manually screened to link TE orders (i.e.: DNA, rolling circle, SINE, LINE, LTR) to unclassified TEs based on characteristic structural features of each order, following a similar approach to (Ray et al. 2019). Finally, we adopted CD-HIT V4.8.1 (Fu et al. 2012), with a sequence identity threshold of 0.80 with available TE libraries for Lepidoptera, to further extend the annotation to remained unknown TEs. All libraries were concatenated without removing redundancies, and RepeatMasker V4.1.4 (Smit et al. 2013) was utilized to re-identify repetitive elements in all the genomes considered in this study.

### Bacterial Contamination and Assembly Completeness Assessment

After the genome assembly contaminants were removed using Blobtools V1.1.1 (Laetsch and Blaxter 2017) using BLASTN [-evalue 1e-25 -max_target_seqs 1] and the NCBI nucleotide collection (#seqs: 49,266,009, retrieved September 2018). Mitochondrial sequences were identified by blasting (BLASTN) and removed from the main assembly. A combination of BUSCO V3.1.0 (Benchmarking Universal Single-Copy Orthologs) (Simão et al. 2015) and the Lepidoptera set in OrthoDB V.10 (odb10) was implemented using default parameters [-m genome], and Exonerate V2.46.2 (Slater and Birney 2005), to assess genome completeness and duplicated content.

### Species Phylogeny and Whole-Genome Alignment

The complete single-copy orthologous genes (scOGs) identified with BUSCO were used to generate the species phylogeny using three Danainae species (*Danaus plexippus, D. melanippus*, and *D. chrysippus*), five Heliconiinae (*Heliconius melpomene*, *H. erato demophoon*, *Eueides isabella*, *Dryas iulia*, *Speyeria mormoria*), the Nymphalinae *Melitaea cinxia,* and two recently available genomes of Ithomiinae (*M. menophilus* and *M. marsaeus rileyi*) (Gauthier et al. 2023). From each locus, the nucleotide sequence was following settings in Couto et al. (2023)Cicconardi et al. (2023, 2017), and Couto et al. (2023), described in more details below. Final alignments were obtained by concatenating single gene alignments and used to estimate the phylogenetic tree using the ML approach as implemented in IQ-Tree2 v2.1.3 COVID-edition (Minh et al. 2020), partitioning the supermatrix for each locus and codon position. IQ-Tree2 was run with the following settings: –runs 5 -m MFP with 5,000 ultrafast bootstrap replicates. As a complement to the ML tree, gene trees from scOGs were using IQ-Tree2 and used to generate a coalescent summary method species tree, as implemented in ASTRAL-III v5.6.3 (Zhang et al. 2018), in order to detect discordant topological signals due to incomplete lineage sorting (ILS).

The Bayesian algorithm of MCMCTree (Yang 2007) was performed adopting the approximate likelihood computation to estimate divergence times, estimating first branch lengths by ML, and then the gradient and Hessian matrix around these calculated in MCMCTree using the DNA supermatrix. Calibration nodes were constrained according to Cicconardi et al. (2023) using a uniform distribution. The analysis was run 10 times each with 100k generations sampled after 10M generations as burn-in, logging every 200 generations. Convergence was checked using Tracer v 1.7.1 (Rambaut and Drummond 2007), verifying values form ESS higher than 200.

For the whole-genome alignment, the ML phylogeny was used to guide the whole-genome alignment using all the previously listed 11 Nymphalid soft-masked genomes, plus the new four new Ithomiini genomes produced by this study. Cactus (Paten et al. 2011; Armstrong et al. 2020) was run using genomes at chromosome level set as the reference.

### Genome Annotations

Raw RNA-seq read data from each library were filtered using Trimmomatic V0.39 (Bolger et al. 2014) (ILLUMINACLIP:$ILLUMINACLIP: 2:30:10; SLIDINGWINDOW: 5:10; MINLEN: 100), and pooled prior to performing the genome annotation. Multiple approaches were adopted (prediction of coding genes, ab initio and de novo) as implemented in the pipeline described in Cicconardi et al. (2023), which maximizes the return from each approach to overcome their own limitations. Briefly, quality filtered reads were mapped using STAR V2.7.10a (Dobin et al. 2013), and the resulting BAM file used as training data for the BRAKER V2.1.5 pipeline (Brůna et al. 2021), which implements GeneMark-ES Suite v4.30 (Lomsadze et al. 2005) and AUGUSTUS V3.4.0 (Stanke et al. 2006). For the de novo transcriptome assemblies, Trinity V2.10.0 (Iyer and Chinnaiyan 2011; Haas et al. 2013) was adopted to generate contigs that were subsequently aligned to the genome using Minimap2. Coordinates for the aligned contigs were used to extract nt sequences, and TransDecoder V5.5.0 (http://transdecoder.github.io/) (minimum amino acid length > 50) was implemented to annotate coding regions, using homologs from the UniProt database (Bateman 2019) and Lepidoptera proteome (see below) found with deltaBLAST V.2.7.1+ (Boratyn et al. 2012); and PFam V33.1 domains (El-Gebali et al. 2019) with HMMscan V3.3.2 (Eddy 1998) (e < 1e−10). To generate the ab initio transcriptomes, BAM files were used as input for both Stringtie V2.1.3b (Pertea et al. 2015) and Cufflinks V2.2.1 (Trapnell et al. 2010, 2012; Garber et al. 2011).

The different annotations (predicted, de novo, and two ab initio) were combined and use with STAR to re-map pooled reads, and Portcullis v1.1.2 (Mapleson et al. 2018) [–threshold 0.5] used to remove false positive spliced sites and to generate a splice-site database. Finally, all these elements (transcript and splice-site annotations) were combined together using Mikado v2.3.3 (Venturini et al. 2018) [–scoring insects.yaml -bt UniprotDB + Lepidoptera –mode split]. To annotate the genome of *G. morgane,* only the prediction pipeline was adopted as RNA-seq data was not available, and only mRNAs and proteins obtained from the other Ithomiini were used as training set for the BRAKER pipeline.

### Identification of Syntenic Single-Copy Ortholog Groups

Finally, the Comparative Annotation Toolkit (CAT) (Fiddes et al. 2018) was used, leveraging the whole-genome alignment, to produce an annotation set on every genome in that alignment, improving gene annotation by identifying previously unannotated loci. The projections of the annotated genes from a reference to the target assemblies was subsequently used with CATgff32OrthologyTable.py, developed in this study, to identify single-copy “syntenic orthologs”, orthologs that follows syntenic information and flanking genomic regions. A similar approached was used by Jebbs et al. (Jebb et al. 2020) in their method TOGA.

### Chemosensory Gene Annotation, Phylogenies of Chemosensory Gene Families, and Orthology Assignment

We inferred the evolutionary relationships of the ORs, IRs, and OBPs annotated from the 14 nymphalid species using amino acid sequences. To do that, we implemented a combination of manual and automatic procedures. We first collected protein sequences from curated datasets available in the literature (Vogt et al. 2015; Bastin-Héline et al. 2019; Yin et al. 2021), which were mapped onto the 14 genomes using Exonerate. From all Exonerate alignments CDS were extracted, and conserved domains were identified, with CD-search (Marchler-Bauer et al. 2015) and PFAM V31 (El-Gebali et al. 2019) using HMMscan (Eddy 2011), combined with the TOPcons web-server (Bernsel et al. 2009; Tsirigos et al. 2015) to identify the presence of the peptide signal, and to predict the number of transmembrane helices (TMHs). At each locus, the best annotation was therefore automatically selected based on the optimal protein length, conserved domain length and score, presence of P-signal and best number of TMHs. Finally, we used the latest version of WebApollo, run using Docker, to manually check the annotations to validate the procedure and correct possible mistakes. Loci with no valid conserved domain hit were then excluded from subsequent analyses (*e* < 1 × 10^−5^). For each chemosensory gene family (CGF), amino acid sequences were aligned using CLUSTALW v2.1 (settings: dnamatrix = IUB; gapopen = 10; gapext = 0.1; gapdist = 10; iteration = TREE; numiter = 1000; clustering = NJ), and the phylogeny was inferred using ML search as implemented in FastTree v2.1.11 SSE3, using Le-Gascuel (2008) model with pseudocounts and the slow exhaustive search algorithm to search for neighbor-joining. For the OR phylogeny, Orco was used as an outgroup; for IR phylogeny, we used the IGluR, and for OBPs, mid-point root. Gene orthology was subsequently assigned based on the phylogenetic tree or reference genes.

### RNA-seq Data Analyses

Quality filtered reads were mapped to the corresponding reference genomes using STAR v2.7.10a [parameters: outSAMattributes NH HI AS NM MD; outFilterMultimapNmax 20; outFilterMismatchNmax 999; outFilterMismatchNoverLmax 0.04; alignIntronMin 20; alignIntronMax 1000000; alignMatesGapMax 500000, alignSJoverhangMin 8; alignSJDBoverhangMin 1; sjdbScore 1]. Expression abundance of each gene/isoform was calculated using RSEM (Li and Dewey 2011) and used as input for intraspecific and interspecific differential expression analysis using EBSeq (Leng et al. 2013) [rsem-run-ebseq], correcting for multiple tests [rsem-control-fdr] with a threshold for posterior probability of 0.95. We also checked for possible bias generating MA plots, PCA and dispersion estimates using normalized counts in DESeq2 implemented in R-project module (Love et al. 2014). For interspecies comparisons TPMs (transcripts per million) were adopted.

Enrichment of GOterms was performed using a combination of two different approaches, the hyperGTest algorithm, implemented in the GOStats package (Falcon and Gentleman 2007) for R [annotation org.Dm.eg.db; conditional TRUE; testdirection over], and GOATOOLS (Klopfenstein et al. 2018) (*P*-value cutoff 0.05); both using the list of expressed genes as the background list. To reduce the false positive rate, *conditional(p)* == TRUE (GOStats) was selected, a conditional algorithm that uses the structure of the GO graph to reduce subsequent tests (Alexa et al. 2006), only considering terms in common between GOStats and GOATOOLS (Klopfenstein et al. 2018) results.

### Selection Dynamics of Chemosensory Genes

For each OG of the three CGFs, the nucleotide sequences were aligned, with a filtering procedure as implemented in (Cicconardi et al. 2023). Briefly, nt sequences were quality filtered before the alignment with PREQUAL v1.02 (Whelan et al. 2018) [-pptype all] and after the alignment, performed with MACSE v2.03 (Ranwez et al. 2011), with HmmCleaner (Di Franco et al. 2019), and GBLOCKS v0.91b (Castresana 2000) under a “relaxed” condition. A ML gene tree was then generated as implemented in IQ-Tree2 v2.1.3 COVID-edition (Minh et al. 2020) [sampling GENESITE; m MFP]. To gain insights into the evolutionary history and the selective pressures on CGFs, we scan for shifts in selective regimes. To do that, we used RELAX (Wertheim et al. 2014) to estimate the selection coefficients (*k*) of orthologous genes for all the chemosensory genes (ORs, IRs and OBPs) in the six Ithomiini species. In brief, RELAX tests whether selection pushes all *ω* categories away from neutrality, intensification, or whereas selection pushes all *ω* categories toward neutrality, relaxation (*ω* = 1). A *k* value is computed to evaluate whether selective strength was relaxed (*k* < 1) or intensified (*k* > 1). We performed the test as implemented in the HYPHY framework (Kosakovsky Pond et al. 2020) to identify genes under intensified selection and test whether different species experience intensification/relaxation for the same genes. To do so, each terminal branch leading to any of the six Ithomiini species was tested using all the other internal and internal branches of the OG as the background. For all selection analyses, the gene tree was used, as a better approximation of gene evolution and history than the species tree (Fukushima and Pollock 2023). All *P*-values associated with *k* were subsequently adjusted for multiple comparisons using Bonferroni correction to be more conservative.

## Supplementary Material

evae218_Supplementary_Data

## Data Availability

Genomic and transcriptomic raw reads together with genome assemblies have been made available via NCBI's Genbank BioProjects: PRJNA1023055, PRJNA1023057, PRJNA1023058, and PRJNA1023059. Details on specific SRA ids can be found in supplementary table S1, Supplementary Material online. Gene annotations and functional annotations are available on Zenodo at the following path: https://zenodo.org/records/13819636. Custom scripts and the updated TE library for Nymphalidae Allfamilies_mod-cdhit.Def.fa is available at https://github.com/francicco/-IthomiineChemosensoryProject.git.
