# Supplementary material for: Novel Sex-Specific Genes and Diverse Interspecific Expression in the Antennal Transcriptomes of Ithomiine Butterflies
Source: Genome Biol Evol. 2024 Oct 7;16(10):evae218. doi: 10.1093/gbe/evae218 (PMC11500719; doi:10.1093/gbe/evae218)
Supplement: evae218_Supplementary_Data [file evae218_supplementary_data.zip › SupplementaryFigures.pdf]

Novel sex-specific genes and diverse interspecific expression in the antennal transcriptomes of ithomiine butterflies

Francesco Cicconardi, Billy J Morris, Jacopo Martelossi, David Ray, Stephen H Montgomery

Supplementary Figures

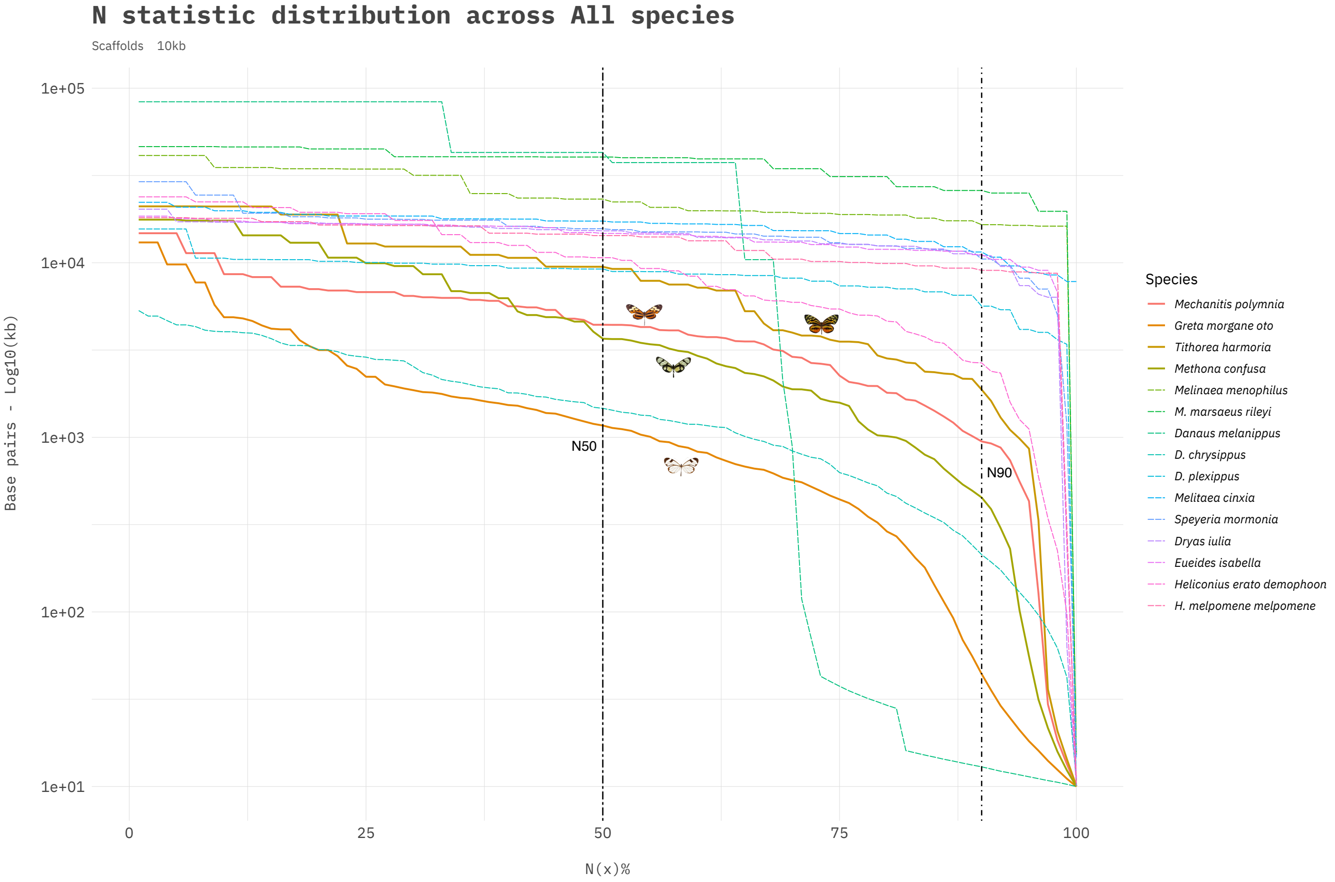

Supplementary Fig. 1 | N(x) distributions across genome assemblies. *The plot shows the contiguity for each genome according to its sequencing/assembly strategy. Dashed lines correspond to already available assemblies, while the solid lines refer to genomes assembled in this study. Note how all assemblies are distributed on the upper side of the plot with a N50 > 1000.*

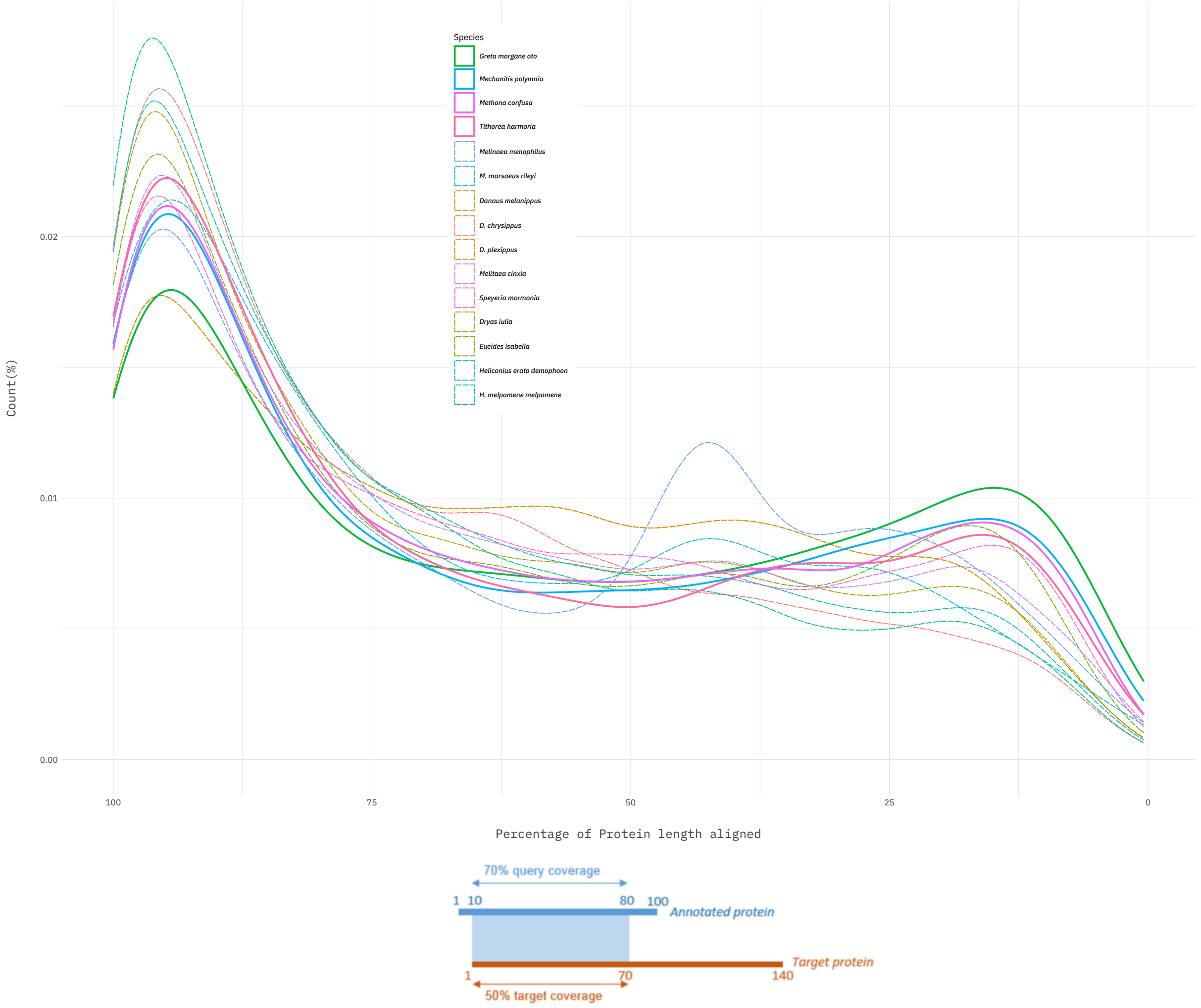

Supplementary Fig. 2 | Transcriptome completeness assessment. *The final set of annotated proteins, which includes a single protein for each locus, was searched with DeltaBLAST against Uniprot DB protein. For each hit the target coverage was calculated as the percentage of the target length that is included in the alignment. The plots show the distribution of the target coverage for each annotation. For almost all annotations the great majority of the transcripts are towards 100% (left side of the x-axes). The target coverage is the percentage of the target length that is included in the alignment. Dashed lines correspond to already annotated genomes, while the solid lines refer to genomes annotated in this study.*

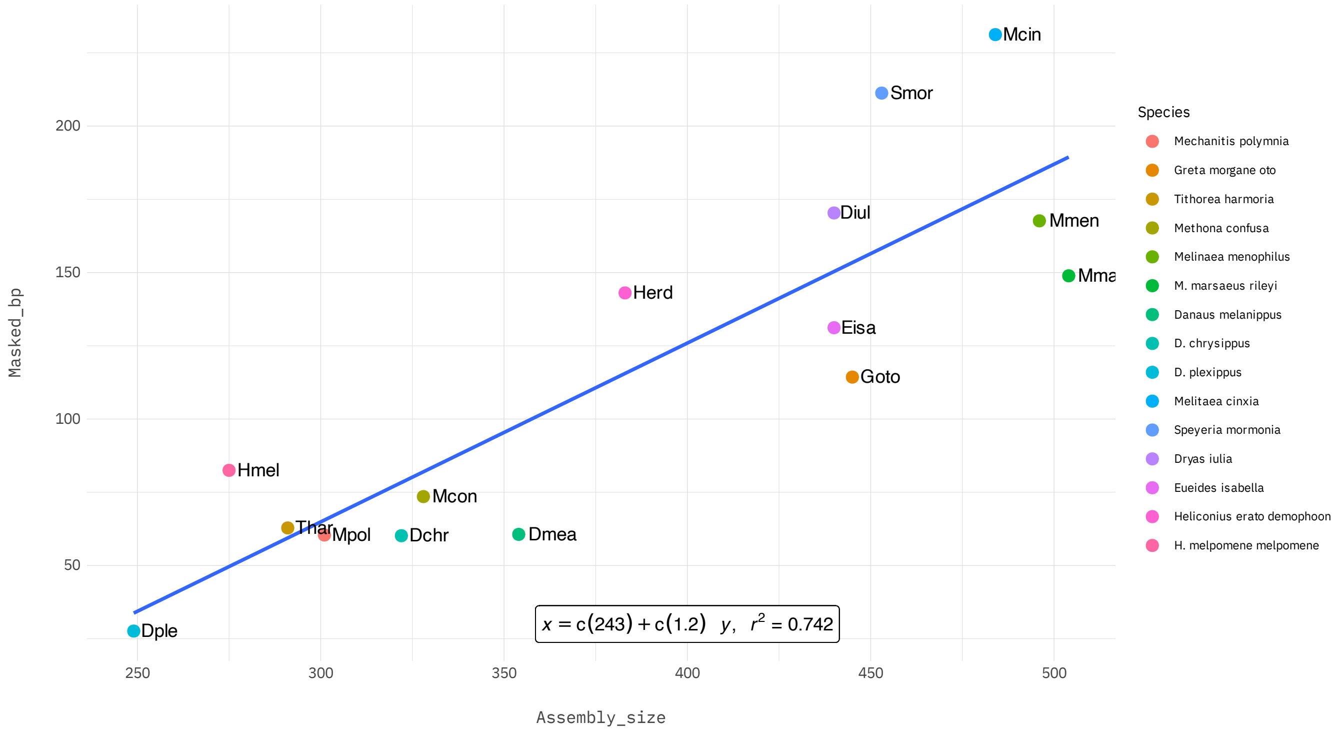

Supplementary Fig. 3 | Transposable Elements vs Genome size*. The relationship between all TEs (masked regions in Mb) and genome size (Mb) indicates a strong correlation between genome size and TE content (Pearson's ρ=0. 86; R^2=0.74).*

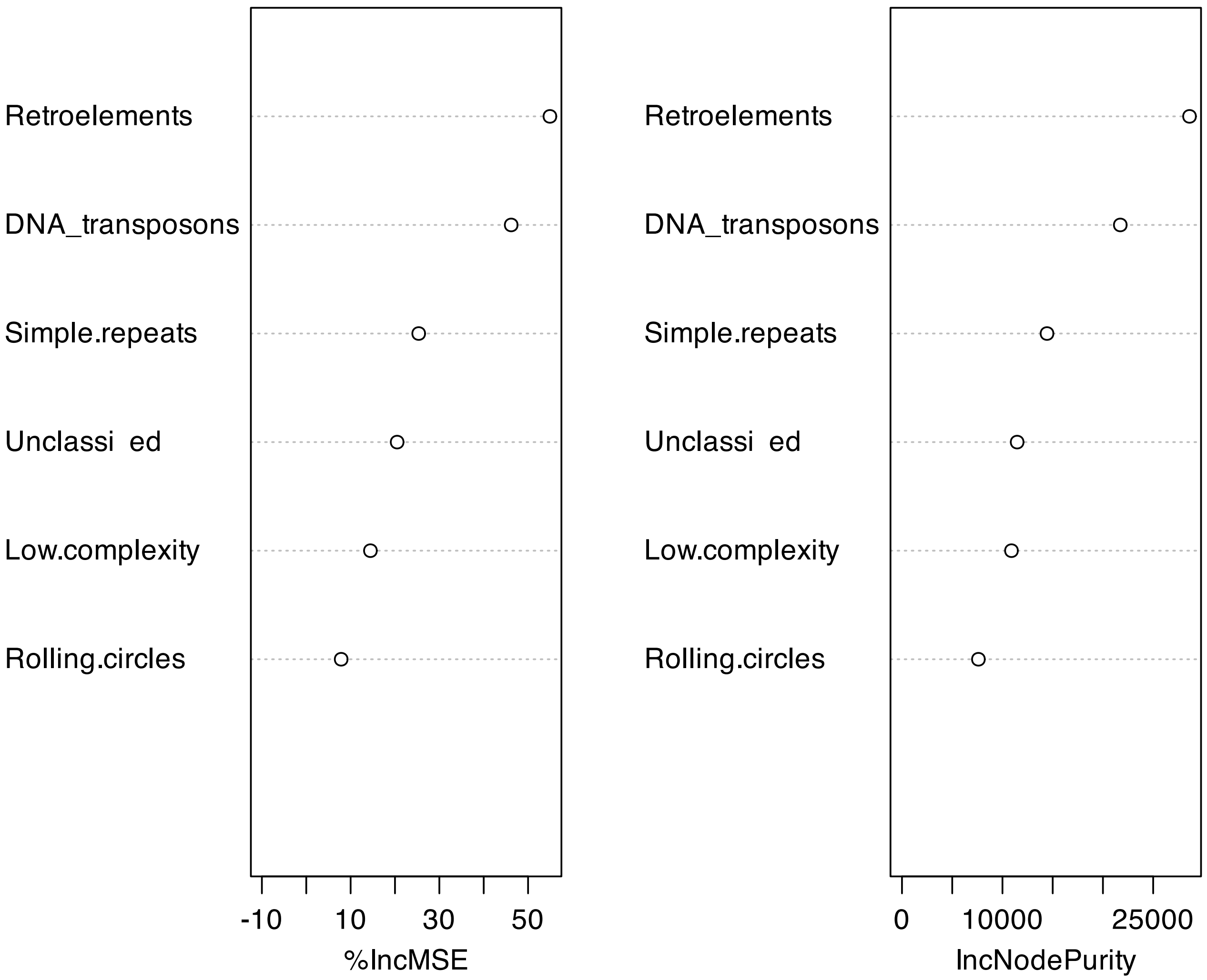

Supplementary Fig. 4 | Main factors affecting genome size. *The plots show random forest analysis (ntree=10,000) using different genomic features. The analysis underlines Retroelements as the feature that is most responsible for variation in genome size, followed by DNA transposons.*

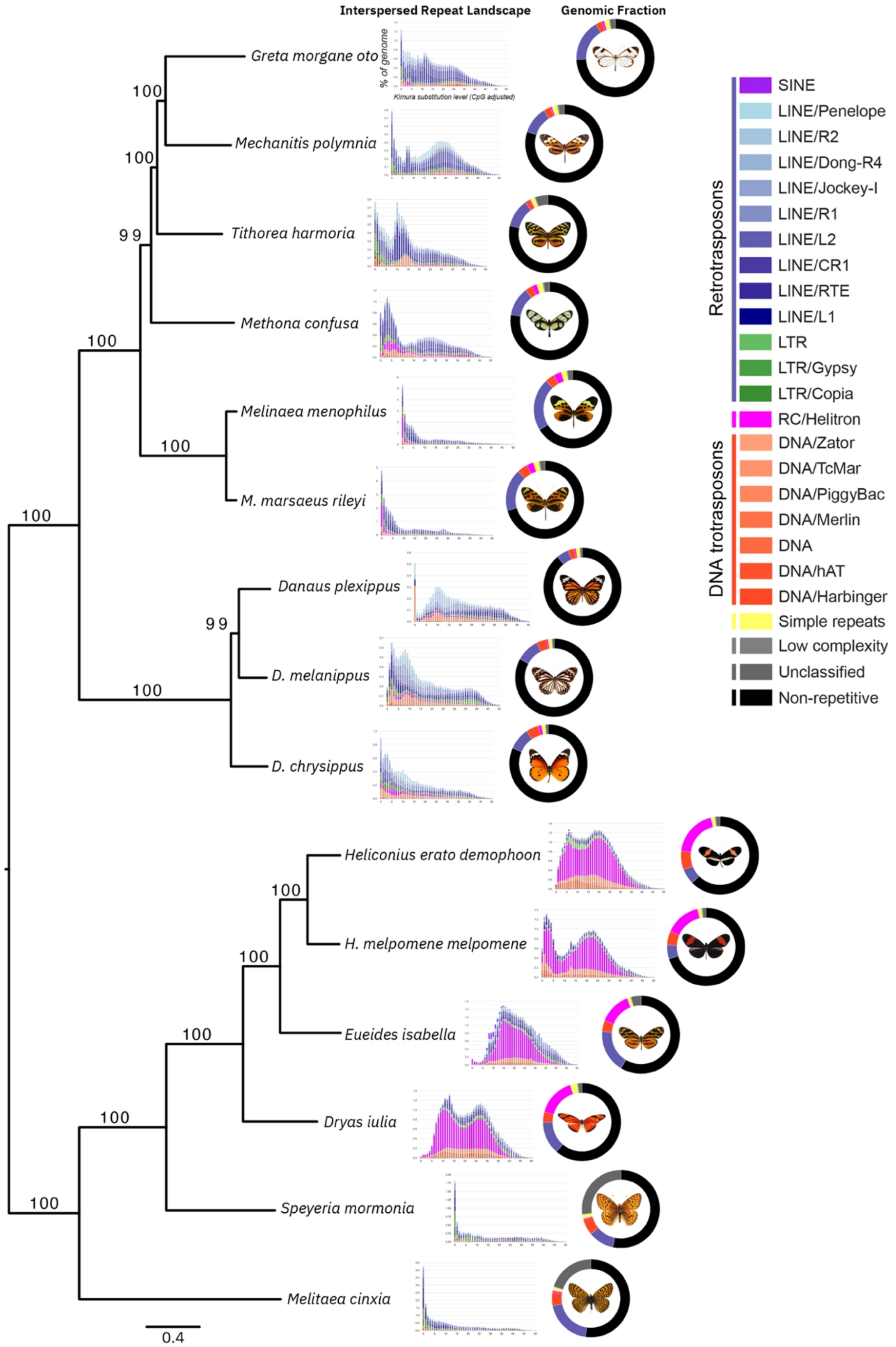

Supplementary Fig. 5 | Maximum Likelihood (ML) phylogenetic tree and TEs landscapes. *ML concatenated species tree of the 15 species taken into account in this study. At each node the corresponding bootstrap value is presented. Next to each species arethe landscape of transposable elements distributions according to the Kimura substitution level (CpG adjusted). Pie charts indicate the fraction of the genome that correspond to the different categories of each element, as indicated in the legend.*

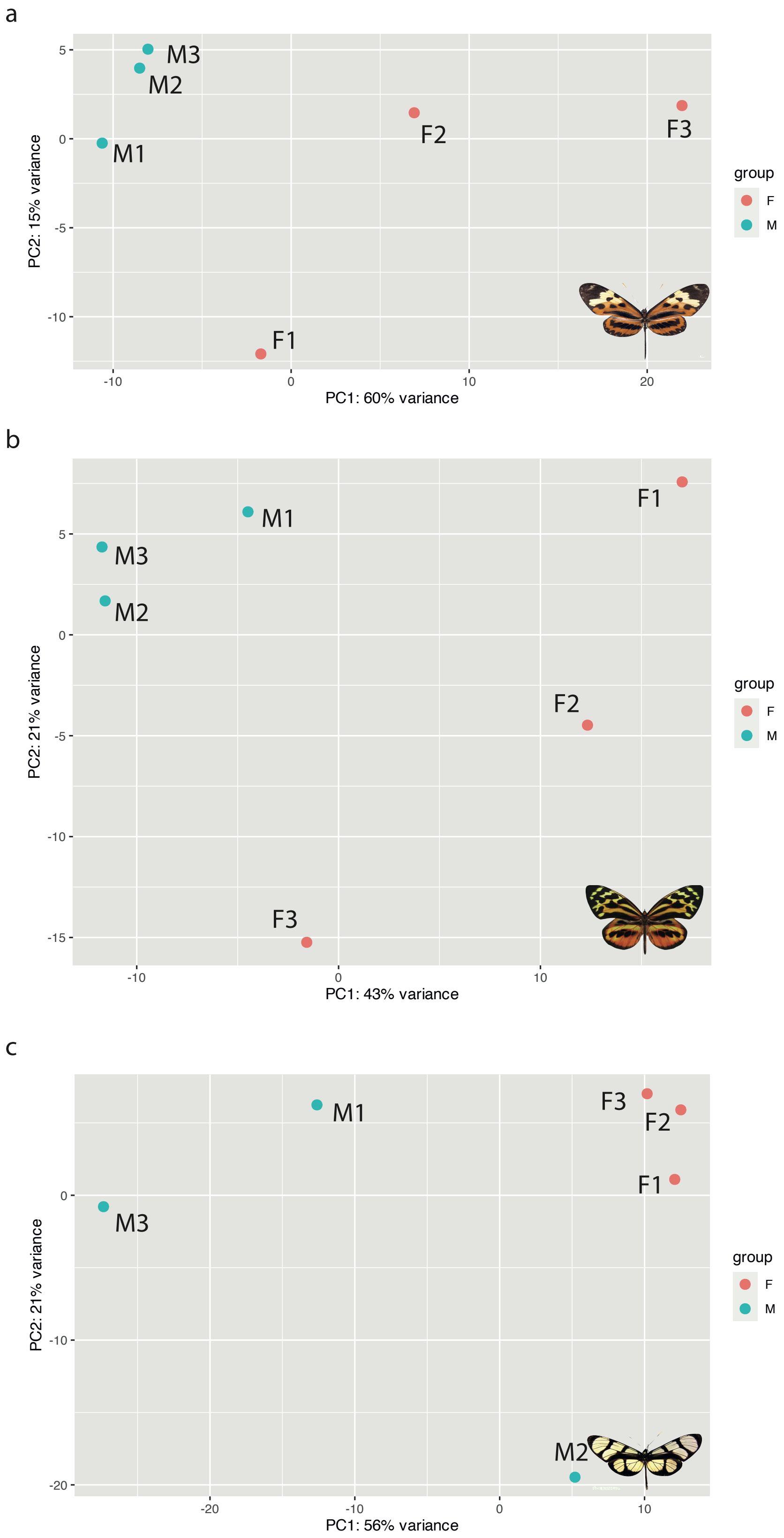

Supplementary Fig. 6 | PCA plots of antennal gene expression. *PCA plots of the number of reads (counts) per gene in the three species. a)* Mc. polymnia; b) T. harmonia; and *c)* Me. confusa.

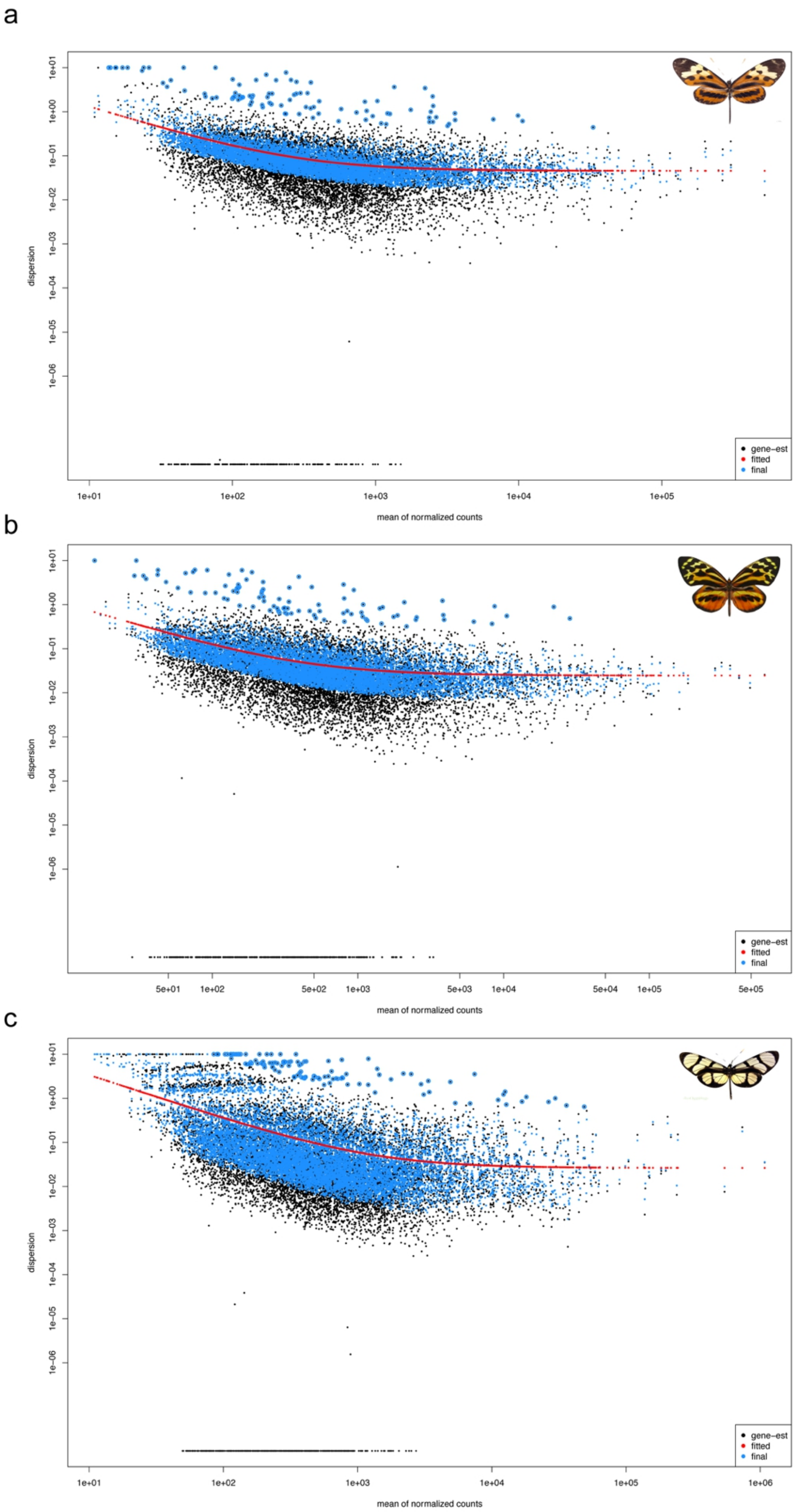

Supplementary Fig. 7 | Dispersion estimate plots. *Each plot shows dispersion (variation) in expression as number of reads (counts) per gene in the three species. Black dots indicate the raw measure; blue dots indicate the normalised values; the red line shows the model of the distribution. The distance from the red line corresponds to the variance of the sample. a)* Mc. polymnia; b) T. harmonia; and *c)* Me. confusa.

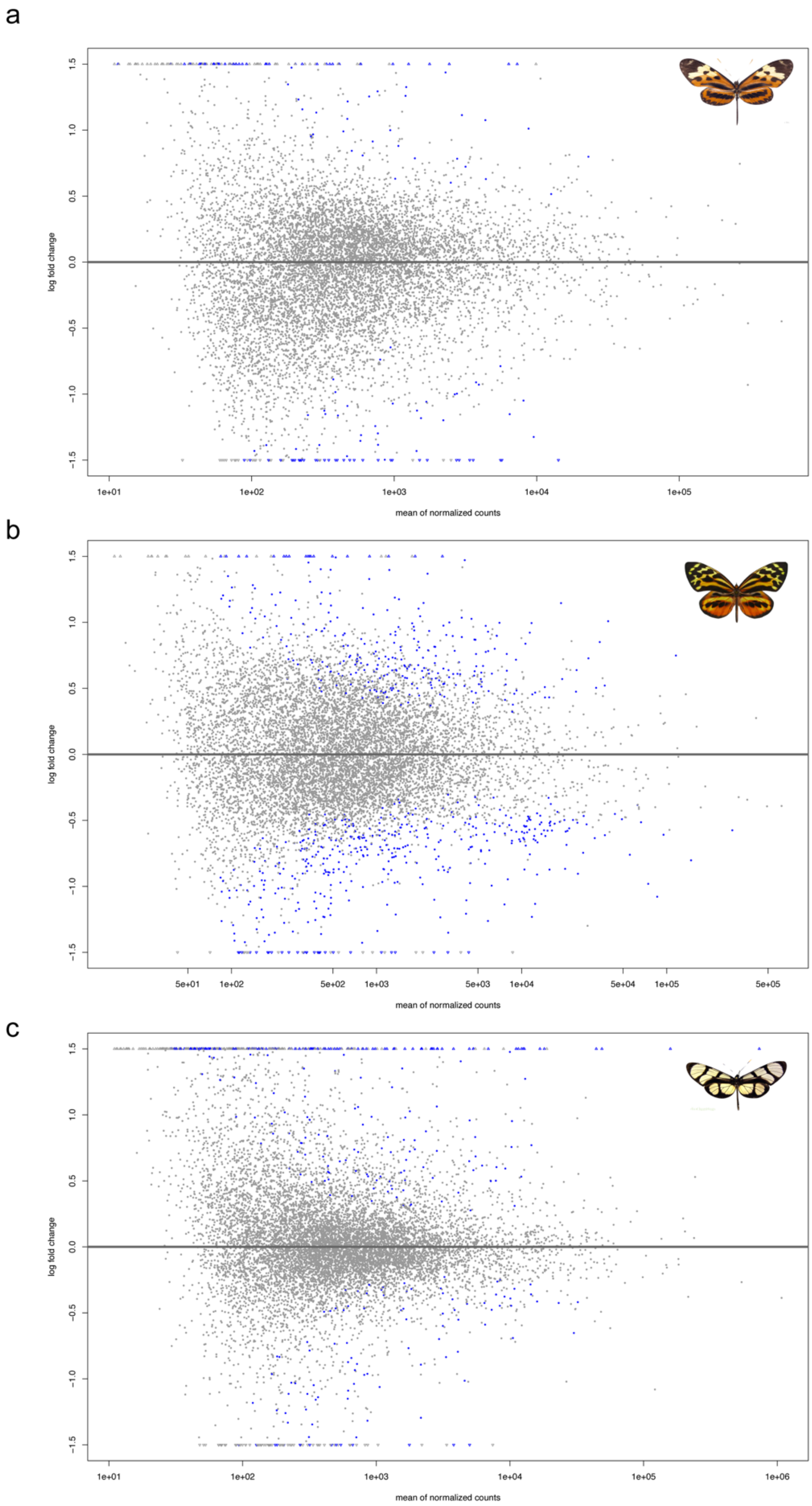

Supplementary Fig. 8 | MA plots. *Each plot shows the dispersion (variation) per gene in terms of fold-change and expression as normalised gene counts. Dots in blue correspond to differentially expressed genes. a)* Mc. polymnia; b) T. harmonia; and *c)* Me. confusa.

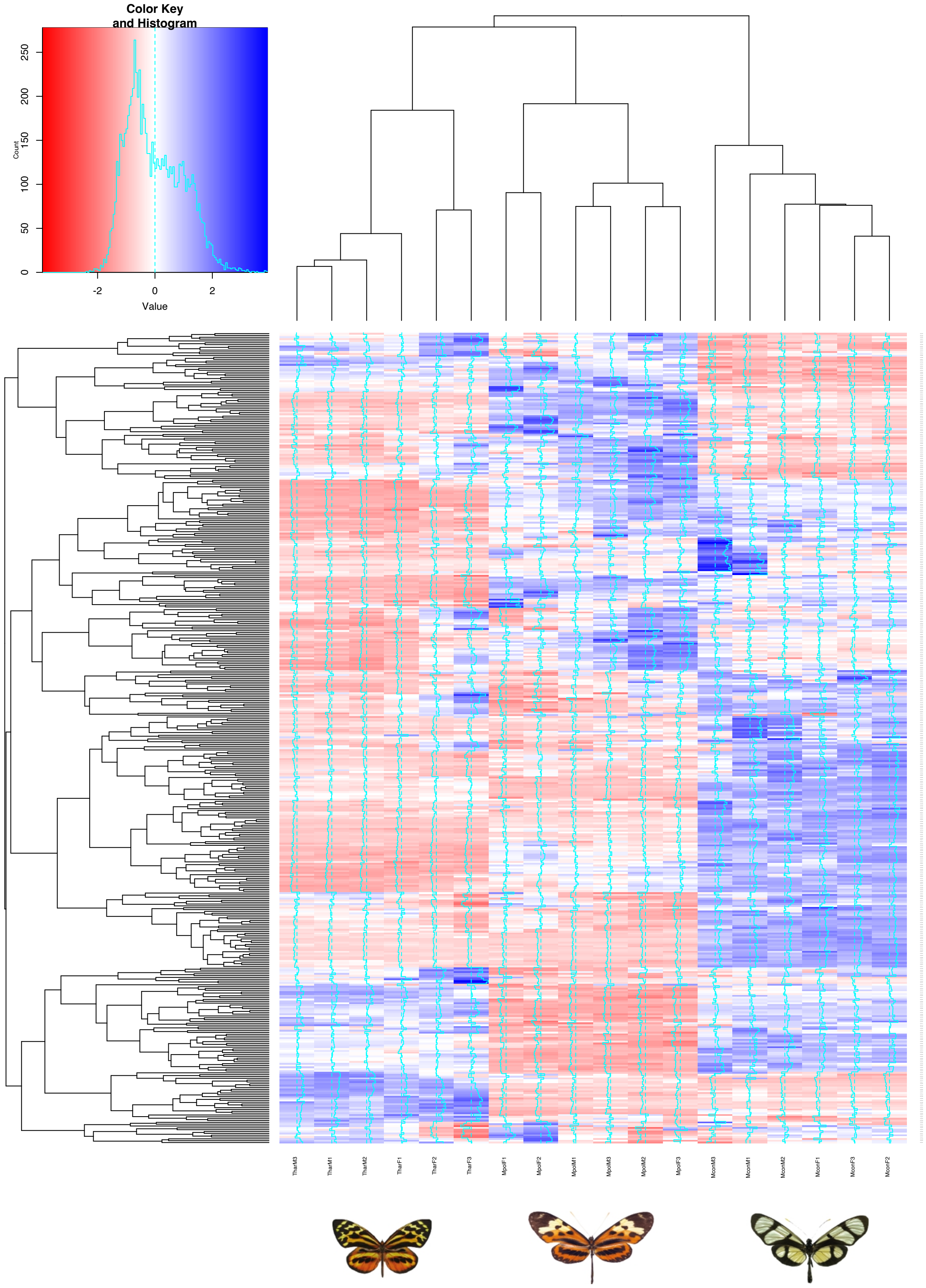

Supplementary Fig. 9 | Heatmap of gene expression among Single-Copy Orthologous Groups. *The heatmap shows the gene expression of single-copy orthologous groups (scOGs) across the three species. Both scOGs and species were clustered based on scOG expression. From left to right all samples from* T. harmonia, Mc. polymnia, and Me. confusa.

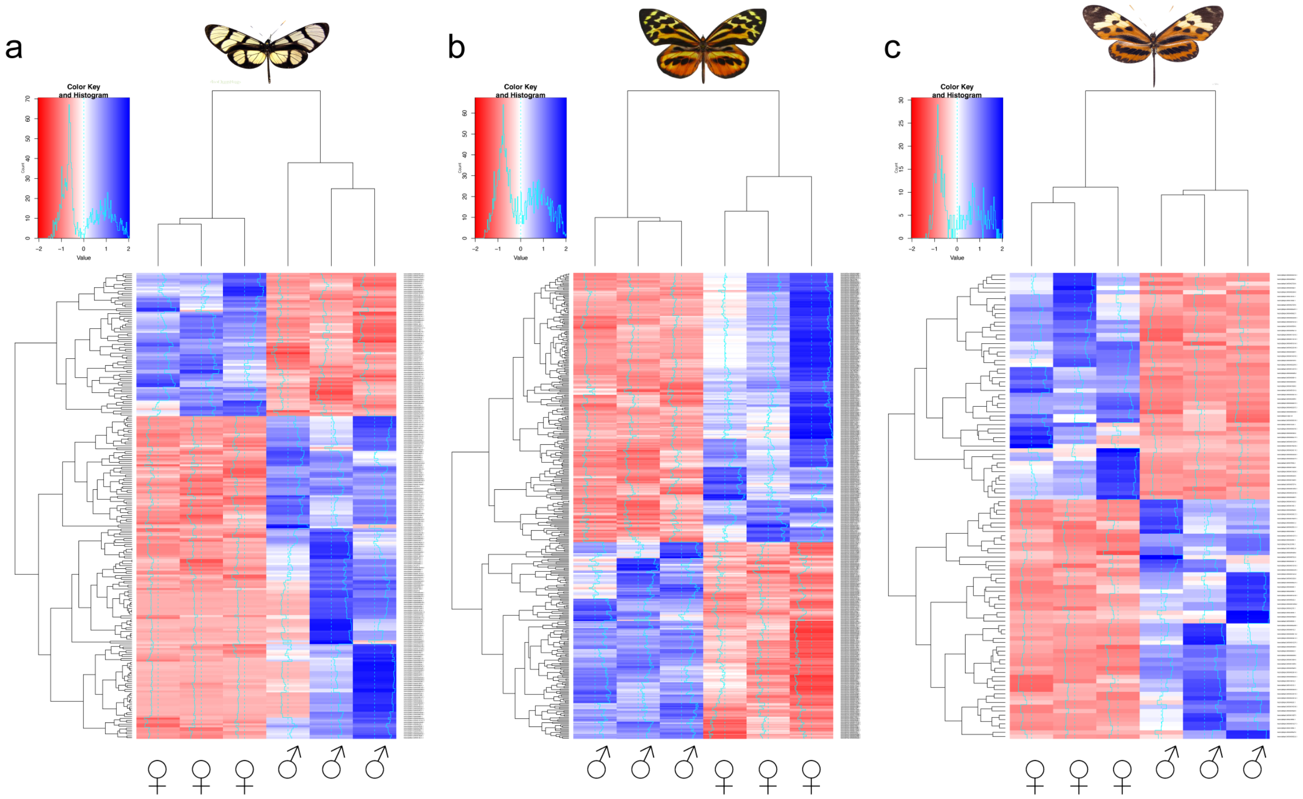

Supplementary Fig. 10 | Heatmap of Differentially Expresed Genes (DEGs) in the three species. *a)* Me. confusa; *b)* T. harmonia*; c)* Mc. polymnia.
